# Student Activity and Sport Study Ireland: Protocol for a Web-Based Survey and Environmental Audit Tool for Assessing the Impact of Multiple Factors on University Students’ Physical Activity

**DOI:** 10.2196/10823

**Published:** 2019-02-21

**Authors:** Joseph J Murphy, Catherine B Woods, Marie H Murphy, Niamh Murphy, Neal Byrne, Ciaran Mac Donncha

**Affiliations:** 1 Department of Physical Education and Sport Sciences University of Limerick Limerick Ireland; 2 Health Research Institute University of Limerick Limerick Ireland; 3 Sport and Exercise Sciences Research Institute Ulster University Newtownabbey United Kingdom; 4 Department of Health Sciences Waterford Institute of Technology Waterford Ireland

**Keywords:** physical activity, universities, students, environment

## Abstract

**Background:**

Increasing proportions of the global population transition through a university setting, a setting associated with engagement in behaviors that diminish health such as high levels of physical inactivity. Increasing physical activity (PA) is a key element of health promotion strategies in many countries, but a better understanding of students’ PA and how it is associated with personal, behavioral, and environmental factors is needed. Studies provide protocols to collect information regarding these factors separately; however, none have developed a validated systematic approach to gather information pertaining to all across a whole country.

**Objective:**

The purpose of this project is to examine students’ physical activity and how it is associated with personal, behavioral, and environmental factors.

**Methods:**

Student Activity and Sport Study Ireland (SASSI) is a university-based cross-sectional study that was carried out across the island of Ireland in 2014. A novel and comprehensive Web-based environmental audit tool (EAT) gathered information pertaining to the environment provided by universities for physical activity. A Web-based student survey (SS) collected information about physical activity beliefs, attitudes, motivations, and behaviors of students. The audit tool and SS were developed through rigorous consultation processes involving international experts. An institutional champion volunteered at each university to recruit, administer, and ensure the completion of both assessments.

**Results:**

Data collection was undertaken between May and December 2014. A total of 80% (33/41) of universities completed the EAT, whereas 88.31% (8122/9197) of students (49.10% [3966/8122] male; mean 23.17 [SD 6.75], years) completed the SS sufficiently. Studies are currently underway with the data collected using this protocol.

**Conclusions:**

SASSI provides a novel and comprehensive protocol for systematically assessing the PA of students and the related personal, behavioral, and *actual* environmental factors. The strengths of the SASSI study are presented and include high response rates and a unique dataset that can provide information to relevant stakeholders and policy makers, along with aiding the development of university environments and interventions that promote PA involvement. The weaknesses of the protocol are recognized with suggestions given to overcome them in future research. This protocol is applicable for other countries and has great potential to create harmonization of data, which would allow for direct comparisons across nations.

**International Registered Report Identifier (IRRID):**

RR1-10.2196/10823

## Introduction

### Background

Early adulthood (ie, ages 18-24 years) is regarded as an exploratory phase, which anchors health-related behaviors that often persist into later life and determine long-term health outcomes [1]. It is becoming increasingly popular for individuals to attend a university during early adulthood. The global student population exceeded 178 million in 2010 and is expected to reach 263 million by 2025 [2]. Research indicates a high proportion of university students engage in behaviors that diminish their health, such as high levels of physical inactivity (23%-44%) [3], and exceeding the daily recommended alcohol and tobacco smoking limits [4]. With students exposed to multiple health-related behaviors of both a positive and negative nature, it seems prudent to focus on a behavior known to benefit the physical, cognitive, and social health of individuals, such as physical activity (PA) [5]. The recommendation to increase PA is a key element of health promotion strategies in many countries [3], where PA includes sport, structured exercise, and active transport [6]. In the general population, PA is an important factor for the prevention of noncommunicable diseases such as obesity, cardiovascular heart diseases, and type 2 diabetes mellitus [7,8]. Although PA levels of children and adults across the globe are well documented [9], university students’ behaviors, beliefs, and attitudes, and how these are formed and reinforced, require further research, particularly in representative or random samples [10].

Understanding the factors that relate to PA is a key step for developing effective evidence-based programs [11]. Social cognitive models have performed well for understanding the factors that relate to individuals’ PA, with Bandura’s social cognitive theory [12] seen as a popular choice for this purpose [13,14]. Social cognitive theory proposes an agentic perspective, suggesting that not only is the environment dictating behavior but also that individuals are being self-regulating and self-developing [12]. Social cognitive theory is founded on a causal model of triadic reciprocal causation in which personal factors, behavioral patterns, and environmental characteristics all interact and influence one another in a bidirectional fashion [12]. However, this theory has been said to focus mainly on the social environment and rarely address the multidimensional role of the physical environment [15].

For this reason, it seems appropriate to also use an ecological approach that summarizes the multiple levels of influence on a behavior, breaking them down into intrapersonal, interpersonal, physical environment, and policy [16]. Furthermore, research has noted the benefits of using social cognitive theory and ecological approaches in combination to investigate the factors relating to PA [17]. Research suggests that personal (eg, age, sex, attitudes, and knowledge of benefits), behavioral (eg, past PA and smoking), and environmental (eg, peer support and recreational PA opportunities) factors are associated with adults and students’ PA [10,18]. Personal and behavioral factors relating to PA are much better understood in university students when compared with research examining associations between PA and the physical environment [10,19,20]. Research is needed to determine and better understand how the environment within which individuals spend time might act to enhance or constrain PA [20]. Our understanding of the impact of the university setting on students’ PA is limited [21], but the physical environment has been shown to influence students’ decision-making process regarding engagement in PA [17]. Nonetheless, evidence is lacking regarding the impact of institution size, support staff, extent and nature of facilities, financial investment, opportunities for participation, and institutional ethos and policy on students’ PA participation.

### Objectives

To date, studies provide protocols to collect both individual [22-24] and environmental information [25,26]; however, none have developed a validated systematic approach to gather information pertaining to the individual (ie, personal and behavioral) and environment across a whole country. Student Activity and Sport Study Ireland (SASSI), the first of its kind, addresses the important topic of the interaction between these factors and participation in PA on the island of Ireland. First, this study aimed to develop and create a comprehensive and usable audit tool for examining the environment, provision, and support offered by universities for students’ PA participation. Second, it aimed to develop a survey to collect information regarding the level, type, and nature of PA participation by students, including the associated determinants, health-related behaviors, and outcomes. Finally, this study aimed to create a protocol, guided by social cognitive theory and an ecological approach that would allow for the audit tool and survey to be used together and provide a holistic view of the factors relating to university students’ PA.

## Methods

### General Information

**Figure 1:**
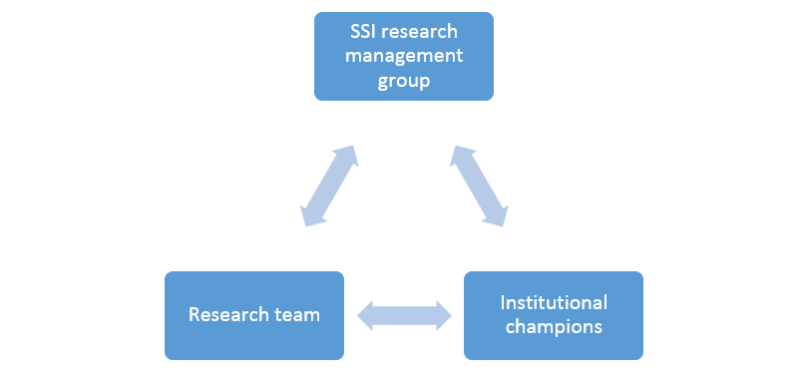
Active partners in Student Activity and Sport Study Ireland. SSI: Student Sport Ireland.

**Figure 2:**
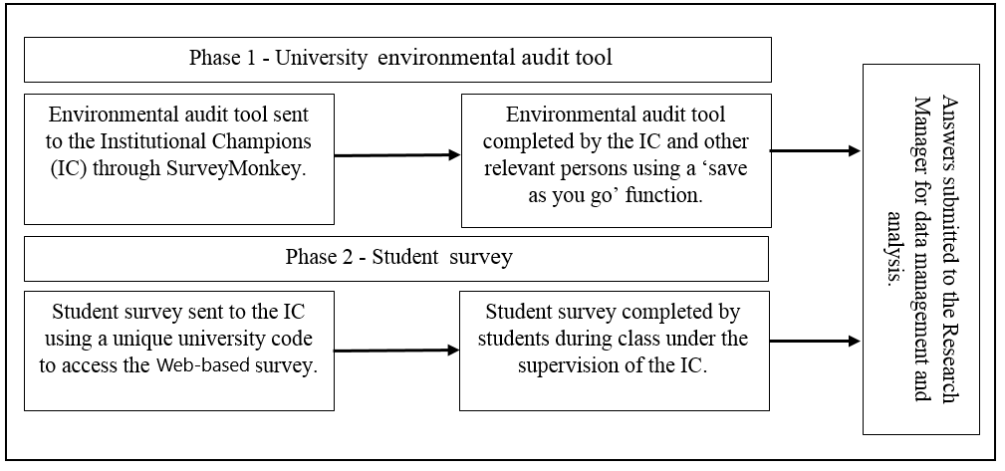
Data collection process for Student Activity and Sport Study Ireland.

Guided by social cognitive theory and an ecological approach, SASSI is a university-based environmental audit tool (EAT) and student survey (SS), which was conducted in 2014. All universities (n=41) on the island of Ireland were invited to partake in SASSI, with universities classified into the following categories, based on their size: (1) large=≥11,000 students; (2) medium=4000 to 10,999 students; and (3) small=≤3999 students. The university size was based on the distribution of the 2013 to 2014 full-time undergraduate and postgraduate enrollment figures across all universities [27,28]. The active partners in the study included the research team, the Student Sport Ireland (SSI) Research Management Group, and the institutional champions (ICs; Figure 1). SSI is the governing body of university sport in Ireland. Owing to the all-island approach, ethical clearance from relevant ethical committees in the Republic of Ireland (Waterford Institute of Technology School of Health Science Research Ethics Committee) and Northern Ireland (Ulster University Research Governance) was obtained and extended through recognition by all universities involved. Detailed information sheets about the study were provided before the start of the EAT and SS. Signed informed institutional consent was received for the EAT, and students were informed that they were providing consent when they chose to proceed with the SS. The data collection process used for the EAT and SS are presented in the coming sections and in Figure 2.

### Institutional Champions

Given the extent of the study and the geographical spread of the universities, the research team relied heavily upon the voluntary contribution of ICs (n=52). In the majority of cases, the IC was the designated contact person for SSI in each of the universities. The ICs were an integral part of the study; their key roles were to promote the research within their university, lead the completion of the EAT through engagement with other institutional stakeholders, and recruit for and administer the SS according to predetermined quotas. To maintain consistency across all universities and ensure the collection of valid data, each IC completed a half-day training program that was used to empower the IC to assist with sufficient data collection. Not only did this process ensure standardization in the implementation across each university, it also created an opportunity to build grassroots commitment and ownership in the study. A research manager was employed as part of the research team and was responsible for overall quality control and ensuring that the ICs were supported in their roles.

### Environmental Audit Tool

The purpose of the EAT was to provide an analysis of the environment and provision made by universities to support student participation in PA. The EAT consisted of eight sections, with 39 questions addressing the following constructs potentially relevant to university support for PA participation: (1) organizational structures; (2) personnel; (3) facilities; (4) funding or investment; (5) opportunities for participation; (6) high-performance athletic support; and (7) institutional ethos, prioritization, and quality of provision. An initial section gathered information on the respondents (eg, title, contact details, and section responsible for completing).

#### Environmental Audit Tool Administration and Completion Procedure

To aid with the distribution and data collection, the EAT was translated into a Web-based instrument using SurveyMonkey (SurveyMonkey Inc, San Mateo, California, USA). The research manager then uploaded the university-specific audit tools, generating a Web-based link for each. This link was sent to the ICs of each participating university via email, who were requested to identify the appropriate personnel in their universities to complete or inform each section. Owing to the time and information required, a *save as you* go function was applied to the EAT, allowing the participants to save answers and return later. This function also allowed the respondents to edit answers before submitting to the research manager.

#### Environmental Audit Tool Development

To guide the development of the EAT, SSI identified the following aspects that should be investigated: (1) local context (eg, location and enrollments); (2) policies and provision; (3) culture (eg, perceived level of support for PA participation); (4) facilities; and (5) needs and resources assessment (eg, current needs and resources to further promote PA). In addition to the above guidance, additional insight into possible content was gained by examining existing literature and other published audits on environments provided by universities in England and Scotland [25,26]. Subsequent to the production of the final EAT, an extensive 6-month consultation process took place to further develop, refine, and confirm it. This included consultation with (1) members of the research team and the SSI research management group (n=10); (2) key stakeholders in the PA provision in universities (n=15, SSI designated contact person); and (3) international experts (n=3, personnel involved in the Sports Provision in Scottish Universities and Irish Higher Education Surveys, and a statistician). These consultations were used to review draft versions of the EAT and maximize face validity of the tools used in the EAT. Face validity is seen as the extent to which a measure appears to provide the desired information and is usually assessed by expert consensus [29]. The whole process resulted in the development of a comprehensive EAT, designed to investigate the environment provided by universities to support and promote PA engagement. An overview of the sections included in the EAT is provided below, with the full version available in Multimedia Appendix 1.

##### Organizational Structure of Physical Activity

To understand the organizational structures of PA within universities, two questions were asked. First, the number of organizational structures (eg, Department of Sport, sport clubs, and student union) which provide direct support to PA participation, the individual (eg, Director of Sport and student services) within the institution that the organizational structure reports to, and a brief description of the role of the structure were asked about. Second, the nature and number of other partnerships within the institution that support sport and PA participation (eg, health service and disability service) were assessed. Responses were open, allowing the respondents to answer from their universities’ perspective.

##### Personnel

The EAT included questions regarding (1) the number of full-time employees, part-time employees, and volunteers supporting PA participation in 2009 and 2014 and (2) the relevant staff titles (eg, *Director of Sport*). This question was answered for each named organizational structure within the university (ie, from previous section). Information regarding training and recognition available to student volunteers was also gathered.

##### Facilities Provision

Questions regarding the extent and nature of both indoor and outdoor facilities available to each university at all locations were included in the EAT. Details about the type of facility (eg, courts and pitches), facility dimensions, specifications and number, ownership (owned or hired), and accessibility for individuals with a disability were gathered. A list of named facilities was included (n=19), and respondents had the option to include *other* facilities. The section included closed responses (ie, yes or no and owned or hired) and open responses to allow more details about the facilities to be provided. Respondents were asked to complete this section for each location used by their university to provide PA opportunities.

##### Funding or Investment for Physical Activity

Investment in PA provision within universities was investigated by gaining insights into the (1) past (last 20 years) and planned (next 5 years) capital investment in facilities by institutional, private, and public sources; (2) current investment in each of the previous 5 years; (3) provision of direct institutional grants for sports clubs; (4) annual fees or charge to students; and (5) student charge to access facility or PA opportunity. Specific funding ranges (eg, up to 25,000; 25,001-35,000) were provided for capital and current investment questions. Open responses were facilitated in the remaining questions.

##### Student Sport and Physical Activity Participation Provision

Questions were asked regarding (1) sports clubs provided by the university; (2) the nature of sports clubs provided (ie, type and provision for individuals with a disability); (3) number of participants; (4) description of link between sports clubs and the universities’ organizational structures; and (5) participation rates in exercise and fitness opportunities. Additional detail was gathered regarding the competition levels engaged in, level of training hours, staffing, income, and expenditure of clubs. A response was requested for a list of 54 named sports clubs (Multimedia Appendix 1), with an option for the respondent to include additional options. The majority of questions were closed in nature, with drop-down menus to facilitate selection of the most appropriate answers.

##### High-Performance Programs and Athletes

Questions regarding various aspects of provision for high-performance programs and athletes were included in the EAT. High performance or elite was defined as students currently competing at national or international standards at either senior or junior levels. The following aspects were examined: (1) institutional partnership with national governing bodies of sport and national or international-level sports clubs and (2) provision, nature, source, and value of athletic scholarships and of *in-kind* athletic support (eg, free access to facilities and sport science support). A combination of open and closed questions was used, and the option of adding *other* choices was included as appropriate.

##### Institutional Ethos and Prioritization

The EAT concluded with questions regarding perceived institutional ethos and prioritization for PA provision. First, respondents were asked about the perceived importance placed on participation in and the promotion of PA and how this importance has changed over the last three years. This was followed by asking about the impact of specific factors (eg, cost of provision and health of students) on the institutional prioritization of PA. Subsequently, the perceived quality of provision under a range of headings (eg, indoor and outdoor facilities, PA opportunities, and funding) for PA was assessed. Finally, the existence and availability of strategic priorities for PA in each university was asked. Likert scales were used to assess the above, with an exception to the final area, which allowed respondents to include a link to any strategic information regarding PA provision.

#### Data Management of the Environmental Audit Tool

The responses from SurveyMonkey (SurveyMonkey Inc, San Mateo, California, USA) were directed to an SPSS database version 22 (SPSS Inc). Each university was given a unique identification (ID) number, which allowed the data to be matched across the EAT and SS. To produce a clean and complete dataset, the following steps were followed: (1) successful data transferal from SurveyMonkey (SurveyMonkey Inc, San Mateo, California, USA) to SPSS was confirmed; (2) missing data were identified and appropriately coded; (3) university size was added; and (4) to ensure that the datasets were anonymous, any text that would enable identification of a specific university was edited. The EAT was developed so that provision for each construct by universities could be usefully scored and analyzed. From the EAT, the following key performance indicators (KPIs) were agreed to represent the environment and provision made by universities to support student participation in PA (Multimedia Appendix 2).

An institutional score for total provision and for provision relative to 100 students was calculated for each KPI listed above. The development of the provision score facilitates analysis of total and relative provision for each KPI across small, medium, and large institutions. In addition, it is also possible to categorize universities as making high, medium, and low provision for each KPI. The different categories of provision were determined by calculating a university rank (1-33) for both the total provision score and the total score relative to 100 students. These two ranking values were then summed and ranked to get a composite rank for each university. On the basis of this composite rank, institutions were assigned equally to either a high, medium, or low provision category for each KPI (ie, ranks 1-11=high; ranks 12-22=medium; and ranks 23-33=low). Details regarding calculation of university total provision score for each KPI are provided in Multimedia Appendix 2.

### Student Survey

The purpose of the SS was to provide information of the students’ behaviors, beliefs, and attitudes regarding sport and PA. The SS consisted of 8 sections, with 98 questions addressing the following areas: (1) general PA; (2) determinants of PA; (3) volunteering in sport; (4) coaching acquired; (5) sport and recreational PA participation; (6) elite athlete satisfaction; and (7) related health behaviors. Additional questions gathered demographic information about the respondent (eg, sex, age, and household income).

#### Student Survey Administration and Completion Procedure

To achieve a nationally representative sample from each university, 3% (7/32) of the student population in large universities, 5% (12/32) of the population in medium-sized universities, and 6% (13/32) of the population in smaller universities were sought. Students were also required from different fields and years of study within each university, depending on the student enrollments [27,28]. Data collection implemented a stratified cluster design for subject selection, stratified by year group and across fields of study, which allowed for a representative sample based on university enrollments. A quota of students needed from each university was developed and given to the IC responsible. The IC then worked alongside the research manager to ensure that the sample was representative of their student body. The IC requested access to the required students and administered the SS during class time, which was completed using the SurveyMonkey software (SurveyMonkey Inc, San Mateo, California, USA). Before the students were given their university-specific survey link, the study was explained, and it was advised that the SS be completed on a laptop, tablet, or mobile phone. The use of a supervised Web-based survey was to maximize response rates, minimize potential for data entry errors, and facilitate the merging of data from over 30 universities. Administering the survey during class time was based on previous research where response rates in excess of 90% have been achieved [3,30]. To ensure that the ICs collected the data as requested, the date stamp of responses was examined by the research manager. Where the majority of responses (>90%) occurred in batches and within normal university hours, it was deemed likely that the protocol was adhered to. The ICs were encouraged to collect as many responses as possible. Where the response rate was greater than the quota needed, the research manager drew a random stratified sample to obtain a representative sample for the overall study. This allowed each university to use their own full dataset for further local analysis while the quota for the national survey was achieved.

#### Student Survey Development

The SS was developed using versions of known tools and measures that have been used in similar studies [23,24,31-33]. The research team consulted with the SSI research management group (n=7), international experts (n=3; health professionals), and statisticians (n=2) to develop and refine the SS through a series of drafts (n=4) over a 5-month period. Again, these consultations were used to review draft versions of the survey, generate consensus among experts, and maximize the face validity of the tools used in the SS. The final SS used open and closed questions to gain the relevant responses, with any sensitive questions related to personal or financial circumstances placed at the end of the survey, as they can be a barrier to further survey completion [34]. Filtering was applied throughout the survey so that the relevant questions were asked based on participants’ previous responses. An overview of the SS’s main sections can be found below, with more information of how the SS was structured, along with the filtering information available in Multimedia Appendix 3.

##### General Physical Activity

Students’ views of their PA levels were asked using five single-item questions, including (1) if they think they take enough PA to keep healthy; (2) their PA levels compared with others; (3) their PA levels compared with those of last year; (4) increasing PA over the next year; and (5) how important PA opportunities were when enrolling. Responses were recorded using a range of Likert scales and categories. Knowledge of the PA guidelines was asked using a single question, with responses allowed in minutes per week or day. General PA levels were measured using three valid and reliable measurement tools for assessing attainment of the PA guidelines [35]: the International Physical Activity Questionnaire-Short Form [36], an adapted version of the *Patient-Centered Assessment and* *Counseling* *for Exercise* [37], and a single-item measure [38]. Domains of PA were measured, including PA as a form of transport, cycling, walking, and muscle strengthening exercises. PA as a form of transport was measured using two questions asking about the form of transport used to get to the university and the duration of time it takes [24]. Students who travelled to the university by a motorized form were asked to give three reasons for not actively travelling, with 12 options available. Walking for recreation was measured with a 3-item question asking about the frequency, duration, and intensity [24]. The frequency and duration of cycling PA [31] and muscle strengthening PA were also assessed using 2-item questions [24].

##### Determinants of Physical Activity

The psychosocial determinants of PA participation were assessed using the Determinants of PA Questionnaire (DPAQ) [39]. Shortened from its original for practical purposes, 1 item for each of the 11 determinants was selected based on the items with the highest factor loading from a confirmatory factor analysis [39]. The shortened DPAQ presents 11 statements, worded positively and negatively, asking students to respond using a 7-point Likert scale ranging from strongly disagree to strongly agree. The determinant areas included knowledge, environmental resources, motivation, beliefs about capabilities, emotion, skills, social influences, beliefs about consequences, action planning, coping planning, and goal conflict related to PA.

##### Volunteering in Sport

A question asked students if they completed any sports voluntary work in the past four weeks, with responses dichotomized into volunteers and nonvolunteers. Those who *volunteered* were asked to indicate the duration (hours per week) and type (range of 7 activities) of volunteering both inside and outside the university.

##### Coaching

This section asked students if they had received any formal coaching or instruction to improve PA performance in the past four weeks, with responses dichotomized into yes or no. If “yes,” then information about where it was accessed was asked with 6 responses provided.

##### Sport and Recreational Physical Activity Participation

Student engagement in recreational PA inside their university was assessed by asking “Did you do any sport or recreational PA in the last four weeks?” with four options that acted as filters, categorizing students as “nonparticipants,” participating only “within university,” “outside university,” or “both in and outside university.” Each category directed to a specific set of questions designed to find out more about their behavioral choices.

Those in the “within university” and “both” categories were asked about the frequency, intensity, duration, standard, and the type of PA they participate in, with options given for each [33]. These students were asked to rate the top five reasons for participation within their university, with 17 responses provided [40], and their satisfaction with provision for PA by their university using 10-point Likert scales. Students were then asked to indicate the uptake of any new PA since beginning in the university and the highest level that they have participated at, through closed questions [41].

Those in the “outside university” category were asked about the frequency, intensity, duration, standard, and the type of PA they participate in, along with whom they participate. The top three reasons for not participating through the university were asked with an option to suggest what their university could do to encourage participation [33]. Questions regarding the reasons for PA participation, the uptake of new activities, and the highest standard participated were then asked.

Those in the “nonparticipants” category were asked for the three reasons for nonparticipation in any PA, the length of time since they last participated, if they could be encouraged to participate in PA (yes or no), and what would encourage them to participate (13 options) both inside and outside the university [26].

##### Elite Athlete Satisfaction

Students who indicated that the highest level participated as “elite” were asked if they received a scholarship or bursary from their university. If “yes,” questions about the sufficiency of scholarship, the type of activities participated in, and their satisfaction with the provision for elite athletes by their university followed.

##### Related Health Behaviors

Questions were asked to assess the health-related behavior choices of students. Alcohol intake, smoking, and drug use were all measured using single-item frequency questionnaires [23]. Sedentary behaviors were measured by students to estimate the minutes spent sitting on weekdays and weekends in a range of 8 situations [42]. Dietary habits were measured using two adapted single-item frequency measures, asking about convenience foods (eg, fast food) and fresh foods (eg, fruit and vegetables) [23]. Students’ perception of body image, general health in the past 12 months, and happiness were assessed using single-item measures with responses recorded using Likert scales [24,31]. Mental health was measured using the 5-item Mental Health Index, a subscale from the Short Form Health Survey [43,44].

#### Data Management of the Student Survey

The sample collected was reviewed against the nationally representative figures once the data collection was complete. This enabled a weighting to be matched to the selection process based on the parameters of age and sex, depending on any gaps or underrepresentation in the initial data collection. The decision to weight by gender and age was based on the knowledge from previous research that participation in sport and PA is significantly influenced by both factors. Weighting of the dataset was completed by statisticians (n=2) and allowed the data to be representative of the national statistics regarding university enrollments. Each dataset was given an ID when data collection was complete, which was the only identifier for each respondent. An ID was also generated based on the university the responses came from, which reflected the ID of the universities in the EAT. This meant the environmental data and SS responses could be matched, allowing examination of the relationship between the university environment and students’ responses to the SS. Reliability of data would affect any future analysis; thus, data cleaning and reliability checks were paramount to this phase. This involved checking data for consistency, completeness, and accuracy through spot checks.

## Results

### Environmental Audit Tool

Data collection using the EAT was undertaken between May and August 2014. A total of 80% (33/41) of universities responded to the EAT. Overall, 70 people from the participating universities played a part in the completion of the EAT, including the following staff or equivalent in each university: Director of Sport; Sports or Clubs and Societies Officer; and Health or PA Promotion Officer. In 42% (14/33) of institutions, the IC only played a role in the completion of the EAT.

### Student Survey

Data collection using the SS was undertaken between October and December 2014. Students from 78% (32/41) of universities participated in this phase of the study. Of the 9197 students administered the SS, 88.31% (8122/9197) provided sufficient responses (49.12% [3966/8122] male; mean 23.17 [SD 6.75] years). Analyses were conducted on the datasets to examine the PA attitudes, beliefs, and behaviors of students and to investigate the influence of relevant factors (ie, individual, behavioral, and environmental) for student PA engagement. The full findings generated from this protocol are available in the SASSI report [45], with additional studies planned in the near future.

## Discussion

### Potential of the Protocol

The SASSI study is a novel, two-phase cross-sectional study combining a purposefully developed EAT and a supervised Web-based SS. Together, the measurement tools provide comprehensive data, which permits an investigation of how personal, behavioral, and environmental factors relate to students’ PA. This enables us to have a holistic view of the factors related to behavior as guided by the social cognitive theory and ecological approach used. In addition, the protocol allows the evaluation of the *actual* environment provided by universities and their association with students’ PA. This has the potential to eliminate the gap in the literature regarding the association of the physical environment with PA engagement in students [10,20]. The data collection tools developed can be used to evaluate existing university provision for PA and measure change in that provision; interrogate and inform the future research agenda; and provide a platform for the pooling and harmonization of data collected. Finally, the authors believe that this protocol is generalizable and can be used in other nations and by other stakeholders to quantify and evaluate the factors that are important for student PA engagement. Such evaluations will contribute to the impact of health promotion efforts for this population.

### Strengths

The protocol has several strengths, which encourage the possibility of its use in other nations wishing to investigate PA in this population. Identification of similar survey instruments and the consultation process throughout the development phases were strengths that allowed for the creation of 2 assessment tools that were used in over 30 universities across 2 nations. The consultation process in both phases also allowed for face validity to be maximized for the EAT and SS by the research team, management group, and international experts. Both the EAT and SS are comprehensive in assessing their intended areas but are designed to be used together, which provide a unique dataset. This unique dataset has the potential to increase our understanding of the *actual* environment provided by universities and the effects it has on students’ PA, while also assessing personal and behavioral factors. Other major strengths of the SASSI protocol concern the training, buy-in, and input from the ICs throughout both phases and the success of administering the EAT and SS through a Web-based platform. These strengths were key factors for the high response rates in the EAT (80% of universities invited completed the EAT) and SS (88.31%; 8122/9197) of the 9197 students administered the SS sufficiently completed it). For phase 2, the use of a supervised survey delivered during class time replicated the response rates of similar study protocols [3,30].

### Limitations

Although the SASSI protocol possesses strengths, the weaknesses also need to be recognized here and addressed for future research. The EAT and SS required a great deal of information and were time-consuming. This magnifies the importance of the buy-in from the ICs and the features offered through Web-based administration (eg, stop-save and ease of administration), which aided completion. Additional limitations concerning the questions used in the EAT and SS need to be addressed. Despite both tools being comprehensive, certain questions and options offered may have resulted in responses that do not provide all the information needed. For example, in the EAT, capital and current investment were assessed using closed questions with the lowest option being “up to €25,000.” This meant that universities that invested €25,000 were grouped with others who invested zero, with no way for the research team to tell the difference. In addition, in the SS, certain health-related behavior questions (eg, alcohol and smoking) assessed frequency but not the intensity of the behavior (eg, units of alcohol or cigarettes). The primary focus of SASSI was PA, which meant less importance was put on other questions, but this is still a limitation of this protocol. Suggestions for future research may be to provide additional options in the closed questions or offer open responses instead. Finally, the validity and reliability of some measures included have not been acquired for this population, with certain measures used in the SS shortened and adapted for practical reasons and to reduce the burden on students. Evidence suggests that lengthy measurement tools lead to greater amounts of missing data on individual questions, decreased variability in answers to grid-based questions, and shorter responses to open-ended questions [46]. Although the methodology allows for face validity of the measures used in the EAT and SS to be maximized through the use of known measures, a consultation phase and training days, there may be a need for future research to test their concurrent validity and reliability. A further weakness of the SASSI protocol was the cross-sectional design that meant the correlates of PA could be measured, but not the true determinants.

### Conclusions

Nevertheless, the authors believe that the SASSI protocol, with its whole country approach, is unique and can be used as a model for other nations hoping to investigate the PA and its related factors in university students. The use of one standardized comprehensive protocol to study such a topic would lead to the harmonization of data, allowing for the comparison of findings across countries. The information collected using the SASSI protocol may have potential uses such as providing information to relevant stakeholders and policy makers, providing strategic guidance for future policy and planning of university settings and university health interventions to enhance the health, well-being, and sustainability of students. The authors also hope that the tools developed in SASSI can be used in future longitudinal research, hoping to investigate the personal, behavioral, and environmental determinants of PA in university students.

## Appendix

Multimedia Appendix 1 Environmental audit tool.

Multimedia Appendix 2 Computation for each key performance indicator stemming from the environmental audit tool responses.

Multimedia Appendix 3 Student survey including the filtering process.

## Abbreviations

DPAQ: Determinants of Physical Activity Questionnaire
EAT: environmental audit tool
IC: institutional champion
ID: identification
KPI: key performance indicator
PA: physical activity
SASSI: Student Activity and Sport Study Ireland
SS: student survey
SSI: Student Sport Ireland
