# Supplementary material for: Student Activity and Sport Study Ireland: Protocol for a Web-Based Survey and Environmental Audit Tool for Assessing the Impact of Multiple Factors on University Students’ Physical Activity
Source: JMIR Res Protoc. 2019 Feb 21;8(2):e10823. doi: 10.2196/10823 (PMC6403525; doi:10.2196/10823)
Supplement: Multimedia Appendix 1 [file resprot_v8i2e10823_app1.pdf]

Supplementary Material 1. Environmental audit tool

**Definitions:**

**Sport -** An activity involving physical exertion and skill in which an individual or team is involved in organised competition against another or others. Sport is governed by a set of rules or customs, which serve to ensure fair competition, and allow consistent adjudication of the winner. Training and preparation for competition is also an aspect of Sport.

**Physical Activity -** Any bodily movement that results in energy expenditure. Physical activity in daily life results from occupational activities, sports activities (non-competitive/recreational), physical conditioning, organised exercise sessions, active transport, or other activities.

## Section 1: Details of Person(s) Completing the Environmental Audit Tool (EAT)

Please provide details of the person(s) completing the EAT in the spaces provided below.

**Q1.** Name of Institution:

| |
|---|
| |

**Q2.** Details of Person(s) Completing EAT

Name:_____________________________ Name:_____________________________

Job Title:___________________________ Job Title:___________________________

Section(s) Completing:_______________ Section(s) Completing:_______________

Telephone (direct dial/ext.):___________ Telephone (direct dial/ext.):___________

Email address:______________________ Email address:______________________

Name:_____________________________ Name:_____________________________

Job Title:___________________________ Job Title:___________________________

Section(s) Completing:_______________ Section(s) Completing:_______________

Telephone (direct dial/ext.):___________ Telephone (direct dial/ext.):___________

Email address:______________________ Email address:______________________

Name:_____________________________ Name:_____________________________

Job Title:___________________________ Job Title:___________________________

Section(s) Completing:_______________ Section(s) Completing:_______________

Telephone (direct dial/ext.):___________ Telephone (direct dial/ext.):___________

Email address:______________________ Email address:_______________

**Section 2: Organisational Structure – Sport & Physical Activity**

**Q1.** Please outline the organisational structure related to sport and physical activity at your institution. One or more structures, which contain multiple levels of administration, may exist. *Please note definitions of sport and physical activity provided in introductory letter above and use the example below as guidance*.

***Example:*** *The following is provided for guidance only. Some colleges may have only two elements in their organisational structure others may have multiple elements of structure.*

| | ***Organisational Structure*** | | ***Who Does Lead Person Report to within Institution*** | | ***Responsibilities. Brief description of role of the structure.*** |
|---|---|---|---|---|---|
| *Structure 1* | *Department of Sport* | | *Director of Finance* | | *All aspects of sport and physical activity provision* |
| *Structure 2* | *Sport and Recreation Service* | | *Student Services Department/Officer* | | *Provides facilities & opportunities of high quality for participation* |
| *Structure 3* | *Athletic or Sports Union or Committee* | | *President Student Union* | | *Organisation, support development of all sport & participation clubs* |
| *Structure 4* | *Campus Company (e.g. Facility management)* | | *Director of Finance Director of Sport* | | *Co-Management of all services and commercial activities at Institution* |
| *Structure 5* | *Sports Clubs* | | *Clubs & Societies Officer* | | *Organisation, support and development of individual sport clubs* |
| *Structure 6* | *Sports Centre of Excellence or Academy* | | *Campus Management Director Senior Executive Sports Manager* | | *Athletic support and services for targeted sports.* |

| | **Organisational Structure** | | **Who Does Lead Person Report to within Institution** | | **Responsibilities. Brief description of role of the structure.** |
|---|---|---|---|---|---|
| Structure 1 | | | | | |
| Structure 2 | | | | | |
| Structure 3 | | | | | |
| Structure 4 | | | | | |
| Structure 5 | | | | | |
| Structure 6 | | | | | |
| Structure 7 | | | | | |
| Structure 8 | | | | | |

**Q2.** Please indicate other structures/departments within your Institution which in partnership support the sport and physical activity provision for students e.g. Buildings/Estates, Health Service, Disability Service, Counselling Service, Careers Service, Student Admissions

| | **Internal Structure/Department** | | **Formal Relationship** | | **Brief description of nature/outcome of the partnership.** |
|---|---|---|---|---|---|

| | | | | | |
|---|---|---|---|---|---|
| Partner 1 | | | Yes ❑ No ❑ | | |
| Partner 2 | | | Yes ❑ No ❑ | | |
| Partner 3 | | | Yes ❑ No ❑ | | |
| Partner 4 | | | Yes ❑ No ❑ | | |
| Partner 5 | | | Yes ❑ No ❑ | | |
| Partner 6 | | | Yes ❑ No ❑ | | |
| Partner 7 | | | Yes ❑ No ❑ | | |
| Partner 8 | | | Yes ❑ No ❑ | | |
| Other | | | Yes ❑ No ❑ | | |
| Other | | | Yes ❑ No ❑ | | |
| Other | | | Yes ❑ No ❑ | | |

**Section 3 – Personnel**

Please use this section to outline the personnel associated with sport and physical activity provision within your institution (full time, part time and voluntary staff). Please use the same description of organisational structure related to sport and physical activity at your institution as presented in Section 2, Q1 above.

**Q1.** Please list the job titles of **all personnel currently** employed in the provision of the sport and physical activity at your institution. Please include all personnel who contribute to provision, regardless of whether this is the main focus of their work. Please indicate number of full, part-time* and volunteer personnel. Additionally please indicate change in personnel numbers since 2009. See example below.

**A part-time employee in Ireland is defined as "an employee whose normal hours of work are less than the normal hours of work of an employee who is a comparable employee in relation to him or her". A comparable employee means a full-time employee to whom a part-time employee compares himself/herself.*

***Example:*** *The following is provided for guidance only. Some colleges may have only two elements in their organisational structure others may have multiple elements of structure.*

| | Organisational Structure | Administrative Structure (Please list job titles supporting sport & physical activity provision) | Total Full Time Staff | | Total Part Time Staff | | Volunteers |
|---|---|---|---|---|---|---|---|
| | | | 2009 | 2014 | 2009 | 2014 | 2014 |
| *Structure 1* | *Department of Sport* | *Director of Sport* | *1* | *1* | | | |
| | | *Administrative staff* | *2* | *2* | *0* | *1* | |
| | | *Secretary* | *1* | *1* | *1* | *0* | |
| | | | | | | | |
| | | | | | | | |
| *Structure 2* | *Sport & Recreation Service* | *Student Services Officer* | *1* | *1* | *1* | *1* | |
| | | *Sports Development Officer* | *1* | *2* | | | *3* |
| | | *Accounts Manager* | *1* | *1* | | | |
| | | *Deputy/Duty Mangers,* | *2* | *3* | *2* | *1* | |
| | | *Administrative staff,* | *2* | *3* | *1* | *2* | |
| | | *Operational Staff,* | *3* | *4* | *4* | *5* | |
| | | *Maintenance staff* | *1* | *2* | | | |

| | | | | | | | |
|---|---|---|---|---|---|---|---|
| *Structure 3* | *Athletic or Sports Union or Committee* | *President Student Union* | *1* | *1* | | | |
| | | *Clubs & Societies Officer* | *3* | *4* | | | |
| | | *Sports Development officers* | *3* | *3* | | | *5* |
| | | *Sports Clubs Secretary,* | *2* | *2* | *1* | *1* | *5* |
| *Structure 4* | *Campus Company (e.g. Facility management)* | *Campus Management Director* | *1* | *1* | | | |
| | | *Accounts Manager,* | *1* | *1* | *0* | *1* | |
| | | *Marketing Manager,* | *1* | *1* | | | |
| | | *Administrative staff* | *1* | *2* | *0* | *1* | |
| | | | | | | | |
| *Structure 5* | *Sports Clubs* | *Chairperson, Secretary (each sport club)* | | | | | *28* |
| | | | | | | | |
| | | | | | | | |
| | | | | | | | |
| | | | | | | | |
| *Structure 6* | *Sports Centre of Excellence or Academy* | *Director* | *0* | *1* | | | |
| | | *Coaches,* | *0* | *2* | | | |
| | | *Exercise Physiologist,* | *0* | *1* | | | |
| | | *Physiotherapist,* | | | *0* | *1* | |
| | | *Administrative staff,* | *0* | *1* | *0* | *1* | |
| | | *Technician,* | *0* | *1* | | | |

| | **Organisational Structure** | **Administrative Structure (Please list job titles supporting sport & physical activity provision)** | **Total Full Time Staff** | | **Total Part Time Staff** | | **Volunteers** |
|---|---|---|---|---|---|---|---|
| | | | **2009** | **2014** | **2009** | **2014** | **2014** |
| Structure 1 | | | | | | | |
| | | | | | | | |
| | | | | | | | |
| | | | | | | | |
| | | | | | | | |
| Structure 2 | | | | | | | |
| | | | | | | | |
| | | | | | | | |
| | | | | | | | |
| | | | | | | | |
| | | | | | | | |
| Structure 3 | | | | | | | |
| | | | | | | | |
| | | | | | | | |
| | | | | | | | |
| | | | | | | | |
| | | | | | | | |
| Structure 4 | | | | | | | |

| | | | | | | | |
|---|---|---|---|---|---|---|---|
| | | | | | | | |
| | | | | | | | |
| | | | | | | | |
| | | | | | | | |
| | | | | | | | |
| Structure 5 | | | | | | | |
| | | | | | | | |
| | | | | | | | |
| | | | | | | | |
| | | | | | | | |
| | | | | | | | |
| Structure 6 | | | | | | | |
| | | | | | | | |
| | | | | | | | |
| | | | | | | | |
| | | | | | | | |
| | | | | | | | |

**Q2.** Please indicate training and recognition available to **student volunteers only** who support sport and physical activity provision at your institution. Please note example in first row below and that the volunteers are registered students

| Volunteer Descriptor | Specific Training Provided | Brief description of training provided | Formal Recognition Provided | Nature of recognition for Volunteering |
|---|---|---|---|---|
| *Example: Coach* | *Yes ✓ No ❑* | *Level 1 accreditation by NGB of sport* | *Yes ✓ No ❑* | *Level 1 Award & Academic Credit Awarded* |
| *Example: Club officer* | *Yes ❑ No ❑* | *Leadership Development* | *Yes ✓ No ❑* | *Leadership Cert* |
| | Yes ❑ No ❑ | | Yes ❑ No ❑ | |
| | Yes ❑ No ❑ | | Yes ❑ No ❑ | |
| | Yes ❑ No ❑ | | Yes ❑ No ❑ | |
| | Yes ❑ No ❑ | | Yes ❑ No ❑ | |
| | Yes ❑ No ❑ | | Yes ❑ No ❑ | |
| | Yes ❑ No ❑ | | Yes ❑ No ❑ | |
| | Yes ❑ No ❑ | | Yes ❑ No ❑ | |
| | Yes ❑ No ❑ | | Yes ❑ No ❑ | |
| | Yes ❑ No ❑ | | Yes ❑ No ❑ | |

**Section 4 – Facilities Provision**

Please use this section to outline the extent of facility provision and access within your institution. Please be as accurate as possible when providing details regarding extent of facility provision. ***Please complete a copy of Question 1 as required for each location (e.g. campus, named facility) so that all sport and physical activity facilities that you have access to are described, state location name below:***

| | |
|---|---|
| Location name: | |

**Q1.** Which of the following indoor and outdoor facilities do you have at this location? Please tick each facility you own / hire and outline the number of courts, pitches, dimensions etc. in the box provided. Please indicate whether there is provision for participation by individuals with disabilities.

| **<u>Indoor Provision</u>** | **Tick if....** | **Tick if….** | 1. Please Provide Details | **Access for Individuals with a Disability** |
|---|---|---|---|---|
| Swimming Pool(s) | Yes ❑<br>No ❑ | ❑ Owned<br>❑ Hired | **25m** \| **33m** \| **50m** | ❑ Yes ❑ No |
| 2. Sports hall(s) | Yes ❑<br>No ❑ | ❑ Owned<br>❑ Hired | No. of badminton courts | ❑ Yes ❑ No |
| Squash courts | Yes ❑<br>No ❑ | ❑ Owned<br>❑ Hired | No. of courts | ❑ Yes ❑ No |
| 3. Handball Courts | Yes ❑<br>No ❑ | ❑ Owned<br>❑ Hired | No. of courts | ❑ Yes ❑ No |
| 4. Fitness suite (CV equipment, | Yes ❑<br>No ❑ | ❑ Owned<br>❑ Hired | No. of stations | ❑ Yes ❑ No |
| 5. Free weights room | Yes ❑<br>No ❑ | ❑ Owned<br>❑ Hired | No. of metres² | ❑ Yes ❑ No |
| 6. Dance / fitness studio | Yes ❑<br>No ❑ | ❑ Owned<br>❑ Hired | No. of metres² | ❑ Yes ❑ No |
| 7. Climbing Wall | Yes ❑<br>No ❑ | ❑ Owned<br>❑ Hired | Wall dimensions | ❑ Yes ❑ No |
| *Other indoor facility (Please Specify)* | ***Tick if....*** | ***Tick if....*** | *Please provide details* | |
| | Yes ❑<br>No ❑ | ❑ Owned<br>❑ Hired | | ❑ Yes ❑ No |
| | Yes ❑<br>No ❑ | ❑ Owned<br>❑ Hired | | ❑ Yes ❑ No |
| | Yes ❑<br>No ❑ | ❑ Owned<br>❑ Hired | | ❑ Yes ❑ No |
| **<u>Outdoor Provision</u>** | **Tick if....** | **Tick if….** | 8. Please Provide Details | **Access for disabled participation** |
| Track & Field | Yes ❑<br>No ❑ | ❑ Owned<br>❑ Hired<br>❑Floodlit | Type of Facilities | ❑ Yes ❑ No |
| 9. Grass Pitch GAA | Yes ❑<br>No ❑ | ❑ Owned<br>❑ Hired<br>❑Floodlit | No of Pitches | ❑ Yes ❑ No |
| Grass Pitch Soccer | Yes ❑<br>No ❑ | ❑ Owned<br>❑ Hired<br>❑Floodlit | No of Pitches | ❑ Yes ❑ No |
| 10. Grass Pitch Rugby | Yes ❑<br>No ❑ | ❑ Owned<br>❑ Hired<br>❑Floodlit | No of Pitches | ❑ Yes ❑ No |
| 11. Synthetic Pitch GAA | Yes ❑<br>No ❑ | ❑ Owned<br>❑ Hired<br>❑Floodlit | No. of Pitches | ❑ Yes ❑ No |

| | | | | | |
|---|---|---|---|---|---|
| 12. Synthetic Pitch Soccer | Yes ❑<br>No ❑ | ❑ Owned<br>❑ Hired<br>❑Floodlit | No. of Pitches | | ❑ Yes ❑ No |
| 13. Synthetic Pitch Rugby | Yes ❑<br>No ❑ | ❑ Owned<br>❑ Hired<br>❑Floodlit | No. of Pitches | | ❑ Yes ❑ No |
| 14. Tennis Courts | Yes ❑<br>No ❑ | ❑ Owned<br>❑ Hired<br>❑Floodlit | No. of Grass | No. of Hard | ❑ Yes ❑ No |
| Access to Walking & Cycling Trails (off road) | Yes ❑<br>No ❑ | ❑ Owned<br>❑ Public<br>❑Floodlit | No of Km's | | ❑ Yes ❑ No |
| Rowing Facilities | Yes ❑<br>No ❑ | ❑ Owned<br>❑ Public<br>❑Floodlit | Type of Facilities | | ❑ Yes ❑ No |
| Sailing Facilities | Yes ❑<br>No ❑ | ❑ Owned<br>❑ Public<br>❑Floodlit | Type of Facilities | | ❑ Yes ❑ No |
| *Other outdoor facility (Please Specify)* | | | *Please provide details* | | |
| | Yes ❑<br>No ❑ | ❑ Owned<br>❑ Hired<br>❑Floodlit | | | ❑ Yes ❑ No |
| | Yes ❑<br>No ❑ | ❑ Owned<br>❑ Hired<br>❑Floodlit | | | ❑ Yes ❑ No |
| | Yes ❑<br>No ❑ | ❑ Owned<br>❑ Hired<br>❑Floodlit | | | ❑ Yes ❑ No |

**Section 5 – Funding for Sport & Physical Activity**

Please use this section to outline the details of past, current and planned investment on sport and physical activity within your institution, in addition please highlight any fees and charges that apply to students for sport and physical activity participation.

**Q1. Capital Investment -** Please outline as accurately as possible the Investment in indoor and outdoor sport and physical activity facilities by institutional or by private or public sources (cost of purchase/development/refurbishment). Please state whether Euros or Pounds (**€/£**).

| | 1995-1999 (€/£) | 2000-2004 (€/£) | 2005-2009 (€/£) | 2010-2014 (€/£) | Planned Investment 2015-2019 (€/£) |
|---|---|---|---|---|---|
| Facilities Indoor | | | | | |

| | | | | | |
|---|---|---|---|---|---|
| Facilities Outdoor | | | | | |

**Q2. Current Investment -** Please outline as accurately as possible the non-capital investment in sport and physical activity (PA) provision by your institution in the past 5 years. (Please try to allocate spending under the headings provided and incorporate as appropriate all current spending e.g. Personnel costs, equipment, capitation or grant funding, etc.).

| | **2009 (€/£)** | **2010 (€/£)** | **2011 (€/£)** | **2012 (€/£)** | **2013 (€/£)** |
|---|---|---|---|---|---|
| Facility Hire | | | | | |
| Sports Clubs (Representative Sport) | | | | | |
| Sports Clubs (PA, Recreational Sport) | | | | | |
| Non-Club Sport (PA, Recreational) | | | | | |
| Exercise and Fitness Programmes (PA) | | | | | |
| Active Commuting Programmes (PA) | | | | | |
| Other (Please Specify) | | | | | |

| | | | |
|---|---|---|---|
| **Q3a.** | Sports Club Capitation Grant - Does your institution provide annual funding (Capitation or Grant) for Sports Club activity? | Yes ❑ | Go to Q3b. |
| | | No ❑ | Go to Q4. |

**Q3b.** If yes, please consider the scenarios below of how the annual capitation/grant is distributed and indicate the scenario **which best fits your** institution.

| **Scenario of Distribution to Sports Clubs** | **Please ✓** |
|---|---|
| Through a Student Union. | ❑ |
| Through a Students Union, Athletic Union or Committee with an input by Sports Department/Sport and Recreation Service/Sports Management. | ❑ |
| Through an Athletic Union or Committee which reports to a Sports Department/ Sport and Recreation Service/Sports Management. | ❑ |
| Through a Sports Department/ Sport and Recreation Service/Sports Management. | ❑ |
| Other (Please Specify) | ❑ |

**Q4.** Please indicate the fees and charges that apply to a) support the provision of sport and physical activity opportunities for students and b) are directly charged to access sport and physical activity opportunities.

| **Type of Levy/Fee** | **Tick if Levy/Fee applies** | **Amount of Levy/Fee (€/£).** |
|---|---|---|
| Compulsory Levy/Fee, all students at registration in addition to normal registration Levy/Fee | Yes ❑ No ❑ | |
| Annual Membership (Access to facilities charge) | Yes ❑ No ❑ | |

| Type of Charge | Tick if Charge Applies | Amount of Charge (€/£). |
|---|---|---|
| Entrance Charge/session or visit to facilities | Yes ❑ No ❑ | |
| Recreation Programme Charge. (Organised Class/Event) | Yes ❑ No ❑ | |
| Exercise Class Charge | Yes ❑ No ❑ | |
| Group Booking Charge (Casual Recreation, Indoor / Outdoor) | Yes ❑ No ❑ | |
| Club Session Charges (Indoor or Outdoor) | Yes ❑ No ❑ | |
| Other (Please Specify) | Yes ❑ No ❑ | |
| | Yes ❑ No ❑ | |

## Section 6 – Student Participation Provision

In this section, please outline the opportunities in your institution for student participation in sports clubs, non-club sport, and exercise or fitness activities.

**Q1.** Please state total number of student sports clubs within the institution (please exclude clubs where there is no physical activity e.g. chess, gaming etc.)?

Insert No. of Sports Clubs in 2013-2014:

**Q2.** For each of the sports listed in table below, please indicate the following:

- Participation of male only, female only or both
- Provision for individuals with disabilities
- If there is a development officer
- The level (s) in which students currently participate in these sports (Select all levels that apply A-F, see definitions below)

*Please use definitions below for additional guidance (ref columns A-G), also note example in first row:*

**A.** Participation: recreational and self-development, where the club provides **ongoing and regular** training for those who may wish to progress through a grading process or just want to play the sport on a casual basis rather than a competitive basis.
**B.** Inter-institutional: where a club competes in Inter-College, Intervarsity or University Championship competition.
**C.** Local/County / Provincial Comp.: where a club competes in non-University competitions / open competitions organised by the respective sport's National Governing Body on a county or regional basis.
**D.** National Comp: where a club competes in non-University competitions / open competitions organised by the respective sport's National Governing Body on a national basis.
**E.** Intl. Student Comp: where a club or club members compete in international student competitions recognised by F.I.S.U. (International University Sports Federation) or E.U.S.A. (European University

Sports Federation). Clubs and club members usually qualify or are selected to represent either College/University or Ireland in these events. Indicate team and/or Individual.
**F.** Intl. Comp: where a club or club members compete in international competitions organised by the European or World Governing Body of the respective sport or event. Clubs and club members usually qualify or are selected to represent either College/University or Ireland in these events. Indicate team and/or Individual.

| **Tick all that Apply** | **Male and/or Female Please ✓** | **Provision for individuals with disabilities Please ✓** | **Full/Part-Time Development Officer? Please ✓** | **Level A. Regular Participation** | **Level B. Inter Institutional** | **Level C. Local County Provincial** | **Level D. National** | **Level E. International Student Comp (Team or Indiv.)** | **Level F. International Comp. (Team or Indiv.)** |
|---|---|---|---|---|---|---|---|---|---|
| *Example* | *❑M ❑F* | *❑Y ❑N* | *✓Y ❑N* | | ✓ | | ✓ | *Team* | *Team* |
| Aikido | ❑ M ❑ F | ❑ Y ❑ N | ❑ Y ❑ N | | | | | | |
| Archery | ❑ M ❑ F | ❑ Y ❑ N | ❑ Y ❑ N | | | | | | |
| Athletics | ❑ M ❑ F | ❑ Y ❑ N | ❑ Y ❑ N | | | | | | |
| Badminton | ❑ M ❑ F | ❑ Y ❑ N | ❑ Y ❑ N | | | | | | |
| Basketball | ❑ M ❑ F | ❑ Y ❑ N | ❑ Y ❑ N | | | | | | |
| Billiards & Snooker | ❑ M ❑ F | ❑ Y ❑ N | ❑ Y ❑ N | | | | | | |
| Boxing | ❑ M ❑ F | ❑ Y ❑ N | ❑ Y ❑ N | | | | | | |
| Canoeing | ❑ M ❑ F | ❑ Y ❑ N | ❑ Y ❑ N | | | | | | |
| **Tick all that Apply** | **Male and/or Female Please ✓** | **Provision for individuals with disabilities Please ✓** | **Full/Part-Time Development Officer? Please ✓** | **Level A. Regular Participation** | **Level B. Inter Institutional** | **Level C. Local County Provincial** | **Level D. National** | **Level E. International Student Comp (Team or Indiv)** | **Level F. International Comp. (Team or Indiv.)** |
| Chinese Martial Arts | ❑ M ❑ F | ❑ Y ❑ N | ❑ Y ❑ N | | | | | | |
| Cricket | ❑ M ❑ F | ❑ Y ❑ N | ❑ Y ❑ N | | | | | | |
| Cycling | ❑ M ❑ F | ❑ Y ❑ N | ❑ Y ❑ N | | | | | | |
| Darts | ❑ M ❑ F | ❑ Y ❑ N | ❑ Y ❑ N | | | | | | |
| Equestrian | ❑ M ❑ F | ❑ Y ❑ N | ❑ Y ❑ N | | | | | | |

| | | | | | | | | | |
|---|---|---|---|---|---|---|---|---|---|
| Soccer (11-a-side) | ❑ M ❑ F | ❑ Y ❑ N | ❑ Y ❑ N | | | | | | |
| Soccer (5-a-side) | ❑ M ❑ F | ❑ Y ❑ N | ❑ Y ❑ N | | | | | | |
| Gaelic Football | ❑ M ❑ F | ❑ Y ❑ N | ❑ Y ❑ N | | | | | | |
| Hurling | ❑ M ❑ F | ❑ Y ❑ N | ❑ Y ❑ N | | | | | | |
| Camogie | ❑ M ❑ F | ❑ Y ❑ N | ❑ Y ❑ N | | | | | | |
| Golf | ❑ M ❑ F | ❑ Y ❑ N | ❑ Y ❑ N | | | | | | |
| Gymnastics | ❑ M ❑ F | ❑ Y ❑ N | ❑ Y ❑ N | | | | | | |
| Handball | ❑ M ❑ F | ❑ Y ❑ N | ❑ Y ❑ N | | | | | | |
| Hill Walking | ❑ M ❑ F | ❑ Y ❑ N | ❑ Y ❑ N | | | | | | |
| Hockey | ❑ M ❑ F | ❑ Y ❑ N | ❑ Y ❑ N | | | | | | |
| Judo | ❑ M ❑ F | ❑ Y ❑ N | ❑ Y ❑ N | | | | | | |
| Karate | ❑ M ❑ F | ❑ Y ❑ N | ❑ Y ❑ N | | | | | | |
| Korfball | ❑ M ❑ F | ❑ Y ❑ N | ❑ Y ❑ N | | | | | | |
| Tennis | ❑ M ❑ F | ❑ Y ❑ N | ❑ Y ❑ N | | | | | | |
| Motor Cycling | ❑ M ❑ F | ❑ Y ❑ N | ❑ Y ❑ N | | | | | | |
| Motor Sports | ❑ M ❑ F | ❑ Y ❑ N | ❑ Y ❑ N | | | | | | |
| Mountaineering | ❑ M ❑ F | ❑ Y ❑ N | ❑ Y ❑ N | | | | | | |
| Netball | ❑ M ❑ F | ❑ Y ❑ N | ❑ Y ❑ N | | | | | | |
| Pool | ❑ M ❑ F | ❑ Y ❑ N | ❑ Y ❑ N | | | | | | |
| Tennis | ❑ M ❑ F | ❑ Y ❑ N | ❑ Y ❑ N | | | | | | |
| Rounders | ❑ M ❑ F | ❑ Y ❑ N | ❑ Y ❑ N | | | | | | |
| Rowing | ❑ M ❑ F | ❑ Y ❑ N | ❑ Y ❑ N | | | | | | |
| Rugby League | ❑ M ❑ F | ❑ Y ❑ N | ❑ Y ❑ N | | | | | | |
| Rugby Union | ❑ M ❑ F | ❑ Y ❑ N | ❑ Y ❑ N | | | | | | |
| Sailing | ❑ M ❑ F | ❑ Y ❑ N | ❑ Y ❑ N | | | | | | |
| Snooker | ❑ M ❑ F | ❑ Y ❑ N | ❑ Y ❑ N | | | | | | |

| | | | | | | | | | |
|---|---|---|---|---|---|---|---|---|---|
| Softball | ❑ M ❑ F | ❑ Y ❑ N | ❑ Y ❑ N | | | | | | |
| Squash | ❑ M ❑ F | ❑ Y ❑ N | ❑ Y ❑ N | | | | | | |
| Sub-aqua | ❑ M ❑ F | ❑ Y ❑ N | ❑ Y ❑ N | | | | | | |
| Surfing | ❑ M ❑ F | ❑ Y ❑ N | ❑ Y ❑ N | | | | | | |
| Swimming | ❑ M ❑ F | ❑ Y ❑ N | ❑ Y ❑ N | | | | | | |
| Table Tennis | ❑ M ❑ F | ❑ Y ❑ N | ❑ Y ❑ N | | | | | | |
| **Tick all that Apply** | **Male and/or Female Please ✓** | **Provision for individuals with disabilities Please ✓** | **Full/Part-Time Development Officer? Please ✓** | **Level A. Regular Participation** | **Level B. Inter Institutional** | **Level C. Local County Provincial** | **Level D. National** | **Level E. International Student Comp (Team or Indiv)** | **Level F. International Comp. (Team or Indiv.)** |
| Tae Kwon do | ❑ M ❑ F | ❑ Y ❑ N | ❑ Y ❑ N | | | | | | |
| Trampolining | ❑ M ❑ F | ❑ Y ❑ N | ❑ Y ❑ N | | | | | | |
| Triathlon | ❑ M ❑ F | ❑ Y ❑ N | ❑ Y ❑ N | | | | | | |
| Tug of War | ❑ M ❑ F | ❑ Y ❑ N | ❑ Y ❑ N | | | | | | |
| Ultimate Frisbee | ❑ M ❑ F | ❑ Y ❑ N | ❑ Y ❑ N | | | | | | |
| Volleyball | ❑ M ❑ F | ❑ Y ❑ N | ❑ Y ❑ N | | | | | | |
| Walking | ❑ M ❑ F | ❑ Y ❑ N | ❑ Y ❑ N | | | | | | |
| Water Polo | ❑ M ❑ F | ❑ Y ❑ N | ❑ Y ❑ N | | | | | | |
| Weightlifting | ❑ M ❑ F | ❑ Y ❑ N | ❑ Y ❑ N | | | | | | |
| Windsurfing | ❑ M ❑ F | ❑ Y ❑ N | ❑ Y ❑ N | | | | | | |
| Please list sports/physical activities not included above | | | | | | | | | |
| | ❑ M ❑ F | ❑ Y ❑ N | ❑ Y ❑ N | | | | | | |
| | ❑ M ❑ F | ❑ Y ❑ N | ❑ Y ❑ N | | | | | | |
| | ❑ M ❑ F | ❑ Y ❑ N | ❑ Y ❑ N | | | | | | |
| | ❑ M ❑ F | ❑ Y ❑ N | ❑ Y ❑ N | | | | | | |
| | ❑ M ❑ F | ❑ Y ❑ N | ❑ Y ❑ N | | | | | | |

| | | | | | | | | | |
|---|---|---|---|---|---|---|---|---|---|
| | ❑ M ❑ F | ❑ Y ❑ N | ❑ Y ❑ N | | | | | | |
| | ❑ M ❑ F | ❑ Y ❑ N | ❑ Y ❑ N | | | | | | |
| | ❑ M ❑ F | ❑ Y ❑ N | ❑ Y ❑ N | | | | | | |
| | ❑ M ❑ F | ❑ Y ❑ N | ❑ Y ❑ N | | | | | | |
| | ❑ M ❑ F | ❑ Y ❑ N | ❑ Y ❑ N | | | | | | |
| | ❑ M ❑ F | ❑ Y ❑ N | ❑ Y ❑ N | | | | | | |
| | ❑ M ❑ F | ❑ Y ❑ N | ❑ Y ❑ N | | | | | | |

**Q3.** For each of the sports listed in table below, please indicate the following:

- Sports Club Category (Select one category only i.e. the primary focus of club)*
- Number of students (Male & Female), hours activity in **ONE Week Mid-Spring Semester**
- Aspects of staffing and support
- Fees/Grants/Expenditure

** Regarding Club Categorisation, please use definition below for guidance, also note example in first row:*

**Category 1**: A recreational and self-development focus where the club provides **ongoing and regular** training for those who may wish to progress through a coaching or learning process or just want to play the sport on a casual rather than a competitive basis, and there is a strong social focus.
**Category 2**: A sport where the main focus of club activity is on the training of club members to attain specific grades or achievement levels rather than competitive activity e.g. Martial Arts, Sub-Aqua, Mountaineering.
**Category 3**: A sport which provides a strong competition basis (generally at a level less than national), with emphasis in many cases on inter-college or intervarsity competition, club may also have a strong recreational and social basis.
**Category 4**: A minority competitive sport which competes at or close to top national level (Fencing, Handball).
**Category 5**: A majority competitive sport in which the relevant College/University team/individuals compete at or close to the top national level (Field games, Basketball).

| | **Club Category* Select one only** | **No. of Students participating in ONE Week Mid-Spring Semester Male/Female** | **Average No of hours/ week Club training and competition** | **No of Institution Staff /Grads involved as volunteers** | **No of regular Coaches in Club** | **Club Membership Fee. 2013/14 €/£** | **Grant to Club from Capitation Income or Institution 2013/14. €/£** | **Total Club Expenditure 2012/13 €/£** |
|---|---|---|---|---|---|---|---|---|
| *Example* | *2* | *M 15 F 10* | *5* | *5* | *1* | *€30* | *€1,500* | *€3,000* |

| | | | | | | | | |
|---|---|---|---|---|---|---|---|---|
| Aikido | | M F | | | | | | |
| Archery | | M F | | | | | | |
| Athletics | | M F | | | | | | |
| Badminton | | M F | | | | | | |
| Basketball | | M F | | | | | | |
| Billiards & Snooker | | M F | | | | | | |
| Boxing | | M F | | | | | | |
| Canoeing | | M F | | | | | | |
| Chinese Martial Arts | | M F | | | | | | |
| Cricket | | M F | | | | | | |
| Cycling | | M F | | | | | | |
| Darts | | M F | | | | | | |
| Equestrian | | M F | | | | | | |
| Soccer (11-a-side) | | M F | | | | | | |
| Soccer (5-a-side) | | M F | | | | | | |
| Gaelic Football | | M F | | | | | | |
| Hurling | | M F | | | | | | |
| Camogie | | M F | | | | | | |
| Golf | | M F | | | | | | |
| Gymnastics | | M F | | | | | | |
| Handball | | M F | | | | | | |
| | **Club Category*** | **No. of Students participating in ONE Week Mid-Spring Semester Male/Female** | **Average No of hours/ week Club training and competition** | **No of Institution Staff /Grads involved as volunteers** | **No of regular Coaches in Club** | **Club Membership Fee. 2013/14 €/£** | **Grant to Club from Capitation Income or Institution 2013/14. €/£** | **Total Club Expenditure 2012/13 €/£** |
| Hill Walking | | M F | | | | | | |
| Hockey | | M F | | | | | | |
| Judo | | M F | | | | | | |

| | | | | | | | | |
|---|---|---|---|---|---|---|---|---|
| Karate | | M F | | | | | | |
| Korfball | | M F | | | | | | |
| Tennis | | M F | | | | | | |
| Motor Cycling | | M F | | | | | | |
| Motor Sports | | M F | | | | | | |
| Mountaineering | | M F | | | | | | |
| Netball | | M F | | | | | | |
| Pool | | M F | | | | | | |
| Tennis | | M F | | | | | | |
| Rounders | | M F | | | | | | |
| Rowing | | M F | | | | | | |
| Rugby League | | M F | | | | | | |
| Rugby Union | | M F | | | | | | |
| Sailing | | M F | | | | | | |
| Snooker | | M F | | | | | | |
| Softball | | M F | | | | | | |
| Squash | | M F | | | | | | |
| Sub-aqua | | M F | | | | | | |
| Surfing | | M F | | | | | | |
| Swimming | | M F | | | | | | |
| Table Tennis | | M F | | | | | | |
| Tae Kwon do | | M F | | | | | | |
| Trampolining | | M F | | | | | | |
| Triathlon | | M F | | | | | | |
| Tug of War | | M F | | | | | | |
| Ultimate Frisbee | | M F | | | | | | |
| Volleyball | | M F | | | | | | |
| Walking | | M F | | | | | | |
| Water Polo | | M F | | | | | | |
| Weightlifting | | M F | | | | | | |
| Windsurfing | | M F | | | | | | |
| Please list sports/physical activities not included above | | | | | | | | |
| | | | | | | | | |
| | | | | | | | | |
| | | | | | | | | |

**Q4.** If a recent (within last 5 years) published report of student sport club structure, activities and details exists are you in a position to email this to the Research Officer for the SASSI project

**or**

Alternatively can you provide a website address for the above information

| | |
|---|---|
| **I will email published information to Research Officer** | Yes ❑<br>No ❑ |
| **Insert web link here:** | |

**Q5.** For all Sports Clubs in your institution, which of the following statements best describes the relationship between the Sports Clubs and the College/Sports Department/Sport and Recreation Service/Sports Management? **Please tick one option only**. If clubs exist which do not fit with the description you have selected for all sports clubs, please insert the club/s name/s opposite a more appropriate description in the "Exception" column.

| **Description of Relationship** | **Tick one only** | **Exception Please insert club name** |
|---|---|---|
| 1. Sports Club Officers plan and organise club affairs largely independently of the Sports Department/Sport and Recreation Service/Sports Management. | ❑ | |
| 2. Sports Club Officers and the Sports Department/Sport and Recreation Service/Sports Management jointly plan and organise club affairs. | ❑ | |
| 3. Sports Department/Sport and Recreation Service/Sports Management plan and organise the Sports Club. | ❑ | |
| 4. Sports Club is planned/organised mainly by a Club Development Officer or Club Coach, who liaises with and reports to the Sports Department/Sport and Recreation Service/Sports Management. | ❑ | |
| 5. Sports Club is planned/organised mainly by a Club Development Officer or Club Coach, who does not report to the Sports Department/Sport and Recreation Service/Sports Management | ❑ | |

**Q6. Exercise & Fitness Sessions:** Please provide participation information for males and females in the following exercise and fitness sessions **during ONE Week in Mid-Spring Semester**

| **Sessions** | **Number of male student participants during ONE Week in Mid-Spring Semester** | **Number of female student participants during ONE Week in Mid-Spring Semester** |
|---|---|---|
| Exercise to Music (including spin) | | |
| Exercise - Circuit training | | |
| Exercise - Weight Training | | |
| Dance | | |
| Other (Please Specify) | | |

| | | |
|---|---|---|
| | | |
| | | |

**Q7. Other Physical Activity Opportunities** (e.g. non-sport club, group physical activity, recreational sport) organised by Sports Department/Sport and Recreation Service or Sports Clubs in 2013. Please provide in the table below details regarding this type of activity, broad categorisation*, participation numbers and whether the activity is a once-off event or is ongoing. See example provided

* Regarding Physical Activity Categorisation please use the following for guidance: 1. Beginner Instruction, 2. Sport/PA for all, 3. League Competition, 4. Tournament, 5. Other (Please Specify).

| **PA Opportunities in 2013 (please list)** | **Indoor or Outdoor** | **PA Category** | **No. of students who participate Male/Female** | **No. of hrs per week.** | **Fee €/£** | **Tick if a) Once-off or b) Ongoing** | **Organised by...** |
|---|---|---|---|---|---|---|---|
| *Example - Campus 10k Run* | *In ❑ Out ✓* | *2* | *M 200 F 250* | *N/A* | *€10* | *a) ✓ b) ❑* | *Rec Service* |
| *Example - Basketball League* | *In ✓ Out ❑* | *3* | *M 45 F 45* | *2hrs* | *0* | *a) ❑ b) ❑* | *Basket Ball Club* |
| | In ❑ Out ❑ | | M F | | | a)❑ b)❑ | |
| | In ❑ Out ❑ | | M F | | | a)❑ b)❑ | |
| | In ❑ Out ❑ | | M F | | | a)❑ b)❑ | |
| | In ❑ Out ❑ | | M F | | | a)❑ b)❑ | |
| | In ❑ Out ❑ | | M F | | | a)❑ b)❑ | |

**Q8.** If published details are available on programmes organized in this way e.g. list of events, tournaments, leagues, etc. are you in a position to email this to the Research Officer for the SASSI project

**or**

Alternatively can you provide a website address for the above information

| | |
|---|---|
| **I will email published information to Research Officer** | Yes ❑<br>No ❑ |
| **Insert web link here:** | |

**Q9.** Non-Club Sport booking of facilities by individuals or groups for recreational usage during ONE Week in Mid-Spring Semester. Please provide details regarding this nature of activity in the table below.

| **Activity (please list)** | **Indoor or Outdoor** | **No. of participants during ONE Week in Mid-Spring Semester Male/Female** | **No. of hours per week.** | **Fee €/£** |
|---|---|---|---|---|
| | In ❑ Out ❑ | M F | | |
| | In ❑ Out ❑ | M F | | |
| | In ❑ Out ❑ | M F | | |

| | | | | |
|---|---|---|---|---|
| | In ❑ Out ❑ | M F | | |
| | In ❑ Out ❑ | M F | | |
| | In ❑ Out ❑ | M F | | |
| | In ❑ Out ❑ | M F | | |

| | | | |
|---|---|---|---|
| **Q10a.** | Is there automatic access to sport and physical activity facilities by all registered students? | Yes ❑ | Go to Q10b. |
| | | No ❑ | Go to Q11a |
| **Q10b.** | If Yes, what % of registered students activate their membership by using facilities at least once in a membership period? | (Drop down menu of choices) | |

| | | | |
|---|---|---|---|
| **Q11a.** | Is there a voluntary charge for membership of facilities? | Yes ❑ | Go to Q11b |
| | | No ❑ | Go to Q12 |
| **Q11b.** | If Yes what % of registered students are members in 2013/14? | (Drop down menu of choices) | |

**Q12.** Which of the following methods are used to publicise sporting opportunities for students?

| Please tick all that apply | Used by the University | Used by Sports Clubs |
|---|---|---|
| Posters in sports facilities | ❑ | ❑ |
| Posters in other university leisure locations | ❑ | ❑ |
| Posters in other university locations | ❑ | ❑ |
| Leaflets in sports facilities | ❑ | ❑ |
| Leaflets distributed to all students | ❑ | ❑ |
| Fresher's packs | ❑ | ❑ |
| Intranet | ❑ | ❑ |
| Internet / external website | ❑ | ❑ |
| Student media (newspaper, radio etc.) | ❑ | ❑ |
| Facebook | ❑ | ❑ |
| Twitter | ❑ | ❑ |
| Other (Please Specify) | ❑ | ❑ |
| | ❑ | ❑ |
| | ❑ | ❑ |
| | ❑ | ❑ |

**Section 7 – High Performance Programmes/Athletes**

High performance / Elite performers are defined as students currently competing at National and/or International standard at either senior or junior levels. Please note this section refers to current student athletes attending your institution only.

**Q1.** Which National Governing Bodies (NGB) do you work with in terms of providing support* to high performance/elite student athletes? Please list all

**Support refers to access to facilities, medical support, sport science support, etc.*

| NGB | Brief Description of Relationship with NGB |
|---|---|
| | |
| | |
| | |
| | |
| | |

**Q2a.** Which of the following organisations do you work with in terms of providing support* to high performance/elite student athletes? *Support refers to access to access to facilities, medical support, sport science support, etc.

| | Tick all that apply |
|---|---|
| Irish Sports Council | ❑ |
| Irish Institute of Sport | ❑ |
| Sport Northern Ireland | ❑ |
| Sports Institute Northern Ireland | ❑ |
| Sport England | ❑ |
| UK Sport | ❑ |
| Other (please specify) | ❑ |
| | ❑ |

**Q2b.** Do you have any partnership/link with an external club, which provides opportunities for students to participate in that sport at national level competition?

| Sport | Male | Female | Brief Description of Relationship |
|---|---|---|---|
| | ❑ | ❑ | |
| | ❑ | ❑ | |
| | ❑ | ❑ | |
| | ❑ | ❑ | |
| | ❑ | ❑ | |

| | | | |
|---|---|---|---|
| | ❑ | ❑ | |
| | ❑ | ❑ | |

| | | | |
|---|---|---|---|
| **Q3a.** | Are sports scholarship/bursaries made available by your institution to students athletes? | Yes ❑ | Go to Q3b |
| | | No ❑ | Go to Q10 |

**Q3b.** Please indicate nature of scholarships awarded.

| | **Please ✓** |
|---|---|
| Yes, International Standard | ❑ |
| Yes, National Standard | ❑ |
| Yes, for other Sports (Please Specify) | ❑ |
| | ❑ |
| | ❑ |
| Yes, NGB funding for specific Sport (Please Specify) | ❑ |
| | ❑ |
| | ❑ |

**Q4.** How many sports scholarships/bursaries were offered to male and female students each year for the last five years? Please indicate total investment each year.

| **Year** | **Males No. of Scholarships** | **Females No. of Scholarships** | **Total Investment €/£** |
|---|---|---|---|
| 2009 | | | |
| 2010 | | | |
| 2011 | | | |
| 2012 | | | |
| 2013 | | | |

**Q5.** In what sports are scholarships/bursaries offered to students and does the institution specifically, target these sports for offers of scholarships?

| **Please list Sports** | **Male Female Please ✓** | | **Target Sport Please ✓** |
|---|---|---|---|
| | ❑ | ❑ | ❑ Yes ❑ No |
| | ❑ | ❑ | ❑ Yes ❑ No |
| | ❑ | ❑ | ❑ Yes ❑ No |
| | ❑ | ❑ | ❑ Yes ❑ No |
| | ❑ | ❑ | ❑ Yes ❑ No |

**Q6.** What were the minimum and maximum values of sports scholarships/bursaries available per student in 2013 (please exclude the value of 'in-kind' benefits from these ranges)?

| **Minimum** | **€/£** | | **Maximum** | **€/£** |
|---|---|---|---|---|

**Q7.** What type of 'in-kind' contributions are available to student athletes who receive a scholarship/bursary. Please tick all that apply

| 15. Contribution | **Tick all that apply** |
|---|---|
| 16. Facility Access | ❑ |
| 17. Sports medicine | ❑ |
| Sports Science | ❑ |
| Physical Conditioning Support | ❑ |
| Physiotherapy | ❑ |
| Nutrition | ❑ |
| Lifestyle management | ❑ |
| Education Modules | ❑ |
| Financial Support | ❑ |
| Flexible Academic Cycle | ❑ |
| Additional academic course related tuition | ❑ |
| Other (Please Specify) | ❑ |
| | ❑ |
| | ❑ |

**Q8.** What is the approximate minimum and maximum values of 'in kind' contributions per talented athlete on scholarship/bursary per year?

| **Minimum** | **€/£** | **Maximum** | **€/£** |
|---|---|---|---|

**Q9.** Does the institution allow scholarship athletes access to programmes with reduced academic requirements (CAO Points or A Level Grades)? ❑ **Yes** ❑ **No**

| **If Yes, how is this decided?** | **Tick all that apply** |
|---|---|
| Interview | ❑ |
| Interview plus assessment of sporting achievement | ❑ |
| Sporting achievement only | ❑ |
| References and sporting achievements | ❑ |
| Other | ❑ |

**Q10.** Does the institution actively recruit student athletes? ❑ **Yes** ❑ **No**

If yes, please use the tick boxes below to indicate the method of recruiting athletes.

| **Method** | **Tick all that apply** |
|---|---|
| Via schools | ❑ |
| Via clubs | ❑ |
| Via organised talent identification sessions | ❑ |
| References and sporting achievements | ❑ |
| National Governing Body links | ❑ |
| Other (Please Specify) | ❑ |
| | ❑ |
| | ❑ |

**Q11.** Please indicate the rank-order of importance of sources of Funding of Sports Scholarships.

Please indicate if funds are not gained from suggested source.

| **Funding Source** | **Rank Importance** | **Not a Source of Funding Please ✓** |
|---|---|---|
| Institutional Funding | | ❑ |
| Corporate Sponsorship | | ❑ |
| Donations | | ❑ |
| Fund Raising | | ❑ |
| NGB Grants | | ❑ |
| Other (Please Specify) | | ❑ |
| | | ❑ |
| | | ❑ |

**Section 8 – Institutional Ethos & Prioritisation for Sport & Physical Activity (please note this section should be completed independently by each person involved in the EAT)**

Based on your responses within this EAT please offer your opinion on the ethos and prioritisation for Sport & Physical Activity within your institution, indicating whether Sports and/or Physical Activity participation is prioritised positively within your institution. All 10 point scales below should be perceived as a percentage scale, progressing towards 100% e.g. 1 = 1-10%, 2 = 11-20%, 3 = 21 – 30%, etc.

**Q1a.** How much **importance** do you feel is placed on **participation in and promotion of** (a) sport and (b) physical activity as a strategic priority by the institution as a whole?

Please indicate below on a scale of 0 to 10, **where 0 means not at all important and 10 means that it is of the highest importance.**

Please keep the distinct **definitions of sport and physical activity** in mind :

**(a) Sport -** An activity involving physical exertion and skill in which an individual or team is involved in organised competition against another or others. Sport is governed by a set of rules or customs, which serve to ensure fair competition, and allow consistent adjudication of the winner. Training and preparation for competition is also an aspect of Sport.

**(b) Physical Activity -** any bodily movement that results in energy expenditure. Physical activity in daily life results from occupational activities, sports activities (non-competitive / recreational), physical conditioning, organised exercise sessions, active transport, or other activities.

| | Not Important | | | | | | | | | Of Highest Importance |
|---|---|---|---|---|---|---|---|---|---|---|
| **Sport** | 0 ❑ | 1 ❑ | 2 ❑ | 3 ❑ | 4 ❑ | 5 ❑ | 6 ❑ | 7 ❑ | 8 ❑ | 9 ❑ | 10 ❑ |
| **Physical Activity** | 0 ❑ | 1 ❑ | 2 ❑ | 3 ❑ | 4 ❑ | 5 ❑ | 6 ❑ | 7 ❑ | 8 ❑ | 9 ❑ | 10 ❑ |

**Q1b.** If possible, provide **three** reasons for your judgement above.

| |
|---|
| |

**Q2.** Please indicate the degree of impact you believe that the factors listed below have on the prioritisation of (a) **sport** and (b) **physical activity** in your institution (where 0 means no impact and 10 means highest impact).

| 1 | **Cost of providing participation opportunities and facilities** | | | | | | | | | | |
|---|---|---|---|---|---|---|---|---|---|---|---|
| | **No Impact<br>Highest Impact** | | | | | | | | | | |
| Sport | 0 ❑ | 1 ❑ | 2 ❑ | 3 ❑ | 4 ❑ | 5 ❑ | 6 ❑ | 7 ❑ | 8 ❑ | 9 ❑ | 10 ❑ |
| Physical Activity | 0 ❑ | 1 ❑ | 2 ❑ | 3 ❑ | 4 ❑ | 5 ❑ | 6 ❑ | 7 ❑ | 8 ❑ | 9 ❑ | 10 ❑ |
| 2 | **Attracting Students** | | | | | | | | | | |
| | **No Impact<br>Highest Impact** | | | | | | | | | | |
| Sport | 0 ❑ | 1 ❑ | 2 ❑ | 3 ❑ | 4 ❑ | 5 ❑ | 6 ❑ | 7 ❑ | 8 ❑ | 9 ❑ | 10 ❑ |
| Physical Activity | 0 ❑ | 1 ❑ | 2 ❑ | 3 ❑ | 4 ❑ | 5 ❑ | 6 ❑ | 7 ❑ | 8 ❑ | 9 ❑ | 10 ❑ |
| 3 | **Health Benefits for Students** | | | | | | | | | | |
| | **No Impact<br>Highest Impact** | | | | | | | | | | |
| Sport | 0 ❑ | 1 ❑ | 2 ❑ | 3 ❑ | 4 ❑ | 5 ❑ | 6 ❑ | 7 ❑ | 8 ❑ | 9 ❑ | 10 ❑ |
| Physical Activity | 0 ❑ | 1 ❑ | 2 ❑ | 3 ❑ | 4 ❑ | 5 ❑ | 6 ❑ | 7 ❑ | 8 ❑ | 9 ❑ | 10 ❑ |
| 4 | **Strengthening relationships with external bodies such as Local Authorities and NGBs** | | | | | | | | | | |
| | **No Impact<br>Highest Impact** | | | | | | | | | | |
| Sport | 0 ❑ | 1 ❑ | 2 ❑ | 3 ❑ | 4 ❑ | 5 ❑ | 6 ❑ | 7 ❑ | 8 ❑ | 9 ❑ | 10 ❑ |
| Physical Activity | 0 ❑ | 1 ❑ | 2 ❑ | 3 ❑ | 4 ❑ | 5 ❑ | 6 ❑ | 7 ❑ | 8 ❑ | 9 ❑ | 10 ❑ |
| 5 | **Positive influence on student academic performance** | | | | | | | | | | |
| | **No Impact<br>Highest Impact** | | | | | | | | | | |
| Sport | 0 ❑ | 1 ❑ | 2 ❑ | 3 ❑ | 4 ❑ | 5 ❑ | 6 ❑ | 7 ❑ | 8 ❑ | 9 ❑ | 10 ❑ |
| Physical Activity | 0 ❑ | 1 ❑ | 2 ❑ | 3 ❑ | 4 ❑ | 5 ❑ | 6 ❑ | 7 ❑ | 8 ❑ | 9 ❑ | 10 ❑ |
| 6 | **Generating revenue for institution** | | | | | | | | | | |
| | **No Impact<br>Highest Impact** | | | | | | | | | | |
| Sport | 0 ❑ | 1 ❑ | 2 ❑ | 3 ❑ | 4 ❑ | 5 ❑ | 6 ❑ | 7 ❑ | 8 ❑ | 9 ❑ | 10 ❑ |
| Physical Activity | 0 ❑ | 1 ❑ | 2 ❑ | 3 ❑ | 4 ❑ | 5 ❑ | 6 ❑ | 7 ❑ | 8 ❑ | 9 ❑ | 10 ❑ |
| 7 | **Raising the profile of the institution** | | | | | | | | | | |
| | **No Impact<br>Highest Impact** | | | | | | | | | | |

| | | | | | | | | | | | |
|---|---|---|---|---|---|---|---|---|---|---|---|
| Sport | 0 ❑ | 1 ❑ | 2 ❑ | 3 ❑ | 4 ❑ | 5 ❑ | 6 ❑ | 7 ❑ | 8 ❑ | 9 ❑ | 10 ❑ |
| Physical Activity | 0 ❑ | 1 ❑ | 2 ❑ | 3 ❑ | 4 ❑ | 5 ❑ | 6 ❑ | 7 ❑ | 8 ❑ | 9 ❑ | 10 ❑ |

**Q3.** Has the importance of (a) sport and (b) physical activity as a strategic priority in the institution increased, decreased or remained the same over the **last 3 years**?

| (a) Sport - Please tick | | (b) Physical Activity - Please tick | |
|---|---|---|---|
| Increased | ❑ 1 | Increased | ❑ 1 |
| Decreased | ❑ 2 | Decreased | ❑ 2 |
| Remained the same | ❑ 3 | Remained the Same | ❑ 3 |
| Don't know | ❑ 4 | Don't know | ❑ 4 |

**Q4a.** Are strategic priorities for (a) sport and (b) physical activity identified by your institution?

| (a) Sport - Please tick | | (b) Physical activity - Please tick | |
|---|---|---|---|
| Yes ❑ | Go to Q4b | Yes ❑ | Go to Q4b |
| No ❑ | Go to Q4c | No ❑ | Go to Q4c |

**Q4b.** If a published statement of strategic priorities for (a) sport and (b) physical activity exists are you in a position to email this to the Research Officer for the SASSI project

**or**

Alternatively can you provide a website address for the statement of priorities

**or**

Briefly state strategic priorities in space provided below e.g. targets for participation in sport and PA, success in inter-varsity competitions, additional facilities, etc.

| | |
|---|---|
| **I will email published statement to Research Officer** | Yes ❑ No ❑ |
| **Insert web link here:** | |
| **Brief Statement of Strategic Priorities:**<br>**Sport:**<br><br>**Physical Activity:** | |

**Q4c.** If published/recorded details are available on the levels of participation in sport and/or physical activity are you in a position to email these findings to the Research Officer for the SASSI project

**or**

Alternatively can you provide a website address for the above information

| **I will email published/recorded information to Research Officer** Yes ❑ No ❑ |
|---|
| **Insert web link here:** |

**Q5.** Can you please consider the following aspects of sport and physical activity provision for students within your institution? Please rate the quality of provision, where 0 means no provision and 10 means excellent provision quality on the scale below. This scale should be perceived as a % scale, progressing towards 100% provision quality.

Please keep the distinct definitions of sport and physical activity in mind:

**(a) Sport -** An activity involving physical exertion and skill in which an individual or team is involved in organised competition against another or others. Sport is governed by a set of rules or customs, which serve to ensure fair competition, and allow consistent adjudication of the winner. Training and preparation for competition is also an aspect of Sport.

**(b) Physical Activity -** Any bodily movement that results in energy expenditure. Physical activity in daily life results from occupational activities, sports activities (non-competitive / recreational), physical conditioning, organised exercise sessions, active transport, or other activities.

| **Aspect of Provision** | 0% | ≤10% | 20% | 30% | 40% | 50% | 60% | 70% | 80% | 90% | 100% |
|---|---|---|---|---|---|---|---|---|---|---|---|
| **Sport** | 0 | 1 | 2 | 3 | 4 | 5 | 6 | 7 | 8 | 9 | 10 |
| Indoor Sport | ❑ | ❑ | ❑ | ❑ | ❑ | ❑ | ❑ | ❑ | ❑ | ❑ | ❑ |
| Outdoor Sport | ❑ | ❑ | ❑ | ❑ | ❑ | ❑ | ❑ | ❑ | ❑ | ❑ | ❑ |
| Sport Clubs for Males | ❑ | ❑ | ❑ | ❑ | ❑ | ❑ | ❑ | ❑ | ❑ | ❑ | ❑ |
| Sport Clubs for Females | ❑ | ❑ | ❑ | ❑ | ❑ | ❑ | ❑ | ❑ | ❑ | ❑ | ❑ |
| Range of Sport Competitions | ❑ | ❑ | ❑ | ❑ | ❑ | ❑ | ❑ | ❑ | ❑ | ❑ | ❑ |
| Non-Competitive Sports Clubs | ❑ | ❑ | ❑ | ❑ | ❑ | ❑ | ❑ | ❑ | ❑ | ❑ | ❑ |
| Sport for individuals with disabilities | ❑ | ❑ | ❑ | ❑ | ❑ | ❑ | ❑ | ❑ | ❑ | ❑ | ❑ |
| Overall Rating of Sport Provision | ❑ | ❑ | ❑ | ❑ | ❑ | ❑ | ❑ | ❑ | ❑ | ❑ | ❑ |
| **Physical Activity** | 0 | 1 | 2 | 3 | 4 | 5 | 6 | 7 | 8 | 9 | 10 |
| Individual Exercise & Fitness Opportunities | ❑ | ❑ | ❑ | ❑ | ❑ | ❑ | ❑ | ❑ | ❑ | ❑ | ❑ |
| Group Exercise & Fitness Opportunities | ❑ | ❑ | ❑ | ❑ | ❑ | ❑ | ❑ | ❑ | ❑ | ❑ | ❑ |
| Facilities that Support Active & Safe Commuting for Students | ❑ | ❑ | ❑ | ❑ | ❑ | ❑ | ❑ | ❑ | ❑ | ❑ | ❑ |
| Physical Activity for individuals with disabilities | ❑ | ❑ | ❑ | ❑ | ❑ | ❑ | ❑ | ❑ | ❑ | ❑ | ❑ |
| Overall Rating of Physical Activity Provision | ❑ | ❑ | ❑ | ❑ | ❑ | ❑ | ❑ | ❑ | ❑ | ❑ | ❑ |
| **Other Aspects of Provision** | 0 | 1 | 2 | 3 | 4 | 5 | 6 | 7 | 8 | 9 | 10 |
| Funding for Sport & Physical Activity | ❑ | ❑ | ❑ | ❑ | ❑ | ❑ | ❑ | ❑ | ❑ | ❑ | ❑ |
| Indoor Facilities for Sport & Physical Activity | ❑ | ❑ | ❑ | ❑ | ❑ | ❑ | ❑ | ❑ | ❑ | ❑ | ❑ |
| Outdoor Facilities for Sport & Physical Activity | ❑ | ❑ | ❑ | ❑ | ❑ | ❑ | ❑ | ❑ | ❑ | ❑ | ❑ |
| Institutional Support for Sport & PA | ❑ | ❑ | ❑ | ❑ | ❑ | ❑ | ❑ | ❑ | ❑ | ❑ | ❑ |
| Staffing for Sport & Physical Activity | ❑ | ❑ | ❑ | ❑ | ❑ | ❑ | ❑ | ❑ | ❑ | ❑ | ❑ |
| Other Issues Please state | ❑ | ❑ | ❑ | ❑ | ❑ | ❑ | ❑ | ❑ | ❑ | ❑ | ❑ |

| | | | | | | | | | | | |
|---|---|---|---|---|---|---|---|---|---|---|---|
| | ❑ | ❑ | ❑ | ❑ | ❑ | ❑ | ❑ | ❑ | ❑ | ❑ | ❑ |
| | ❑ | ❑ | ❑ | ❑ | ❑ | ❑ | ❑ | ❑ | ❑ | ❑ | ❑ |
| | ❑ | ❑ | ❑ | ❑ | ❑ | ❑ | ❑ | ❑ | ❑ | ❑ | ❑ |

Thank you for taking the time to complete this Environmental Audit Tool.
All information provided will be treated with the strictest of confidence.
Appropriate security measures will be taken to prevent unauthorised access to all information supplied.
