# Supplementary material for: Student Activity and Sport Study Ireland: Protocol for a Web-Based Survey and Environmental Audit Tool for Assessing the Impact of Multiple Factors on University Students’ Physical Activity
Source: JMIR Res Protoc. 2019 Feb 21;8(2):e10823. doi: 10.2196/10823 (PMC6403525; doi:10.2196/10823)
Supplement: Multimedia Appendix 2 [file resprot_v8i2e10823_app2.pdf]

Supplementary Material 2. Computation for each key performance indicator (KPI) stemming from the environmental audit tool (EAT) responses.

KPI	Relevant question from the EAT, provision score computations and recoding
Organisational Structures	Section 2 - Q1 and Q2; Number of structures and partnerships reported. Total provision score: sum of number of organisational structures (Q1) and internal partnerships (Q2).
Number of Full-time and Part-time staff and volunteers (past year)	Section 3 – Q1; Total number of full time, part time and volunteers report under each organisational structure.<br>Total provision score: Number of full-time staff reported (Weighted x1) + part-time staff (Weighted x0.5) + volunteers (Weighted x0.2). The weighting coefficients represented probable resource implications for the institution (i.e. full-time staff greatest resource implication etc.).
Indoor facilities represented as m^2 provision accessible at primary location and all other owned facilities.	Section 4 – Q1; Number of courts/swimming pools and associated dimensions – only facilities owned or hired at primary location and owned at other locations. Total provision score: m^2 total score was calculated based on specific information provided by respondent or the following national and international recognised dimensions for the relevant facility.<br>Swimming Pool (Weighted x 2): 50m = 1250m2 (50X25m), 33m = 495m2 (33 X 15m), 25m = 375m2 (25x15m), 20m = 200m2 (20 x 10m), 18m = 180m2 (18 x 10m).<br>Sports Hall (no. of Badminton Courts): Badminton Court = 124.74m2 (15.4 x 8.1m)<br>Squash Court = 62.4m2 (9.75 x 6.4m). Handball Court = 167.2m^2 (18.29 x 9.14m)<br>Fitness Suite: Each Station = 5m^2 (2.5 x 2m). Weights Room, Dance/Fitness Studio, Climbing Wall = m^2 provided by respondent.
Outdoor facilities represented as m^2 provision accessible at primary location and all other owned facilities.	Section 4 – Q1; Number of pitches, nature of facility and associated dimensions, only facilities owned or hired at primary location and owned at other locations. Total provision score: m^2 total score was calculated based on specific information provided by respondent or the following national and international recognised dimensions for the relevant facility.<br>Track & Field: 400m Track & Field = 16,373m^2 (177 x 92.5m), Sprint Track – Distance x No of Lanes, Throwing Area = 5000m^2 (100x50m). GAA Pitch (Grass & Synthetic) = 10,400m2 (130x80m). Soccer Pitch (Grass & Synthetic) = 6,500m2 (100 x 65m), Synthetic 5-a-side – 1,750m2 (50 x 35m). Rugby (Grass

	& Synthetic) = 8,400m2 (120 x 70). Tennis Court = 312m^2 (26 x 12m). Trails = Distance in km.
Capital Investment in Indoor and Outdoor facilities (Total since 1995).	Section 5 – Q1; 14 possible investment ranges could be selected. Mid-points of the ranges were used to indicate investment made with the exception of the first and last range where the maximum value was used.<br>Total provision score: Total indoor and outdoor capital investment made since 1995
Total Current Investment (past 5 years)	Section 5 – Q2; 14 possible investment ranges could be selected. Mid-points of the ranges were used to indicate investment made with the exception of the first and last range where the maximum value was used.<br>Total provision score: Combined score of total investment for the past 5 years across: 1) facility hire; 2) representative sports clubs; 3) physical activity, recreational sport clubs; 4) Non-Club Sport; 5) Exercise and fitness programmes and 6) active commuting programmes.
Sport Clubs Participation – Number of sports clubs and total student numbers participating.	Section 6 – Q1; Total number of sports clubs reported. Total provision score: Number of clubs reported.<br>Section 6 – Q3; Number of male and female students participating in specific sport club during one Week Mid-Spring Semester. Respondents were asked to select a specific range which best represented male and female participation, the mid-point of each range was then used to represent the number of participants in each club. The minimum was 0 and the max was 500. Total provision score: Number of participants reported across all clubs.
Exercise & Fitness Participation – Total student numbers participating.	Section 6 – Q6; Number of male and female students participating in a range of exercise and fitness opportunities during one Week Mid-Spring Semester. Respondents were asked to select a specific range which best represented male and female participation, the mid-point of each range was then used to represent the number of participants in each club. The minimum was 0 and the max was 500. Total provision score: Number of participants reported across all activities.
Institutional Ethos and Perceived Quality of Provision for both Sport and PA	Section 8 – Q1a and Q5. Perception of quality of provision for both sport and PA. The total of scores (1-10) allocated to 14 different questions constituting the sport perception score and the total of 11 questions constituting the PA score. Targeted questions were: 1) strategic importance allocated to sport/ PA (Q1a); 2) For Sport only - perceived quality of provision for indoor/outdoor sport, sport club provision, range of sport competitions, non-competitive sports, provision for individuals with a disability and overall rating for sport provision (Q5); 3) For PA only - individual and group PA opportunities, active commuting opportunities,

	provision for individuals with a disability and overall rating for PA provision (Q5); and 4) Provision for both sport and PA regarding funding, facilities, staffing and perceived institutional support (Q5). The total score was then calculated as a percentage of the possible max score for sport, which was 140 and for PA was 110. This value represented the institution score for perceived quality of provision.
