# Supplementary material for: Student Activity and Sport Study Ireland: Protocol for a Web-Based Survey and Environmental Audit Tool for Assessing the Impact of Multiple Factors on University Students’ Physical Activity
Source: JMIR Res Protoc. 2019 Feb 21;8(2):e10823. doi: 10.2196/10823 (PMC6403525; doi:10.2196/10823)
Supplement: Multimedia Appendix 3 [file resprot_v8i2e10823_app3.pdf]

Supplementary Material 3. Student Survey including the filtering process.

No.	Question - Origin	Question	Responses			Psychometric Information (if available)
**Background Information**						
1*	N/A	Are you over 18? (Consent)	Yes (continue)	No (finish)		N/A
2	N/A	Year and Month of Birth	Year	Month		Face Validity
3	N/A	Are you Male or Female	Male	Female		Face Validity
4*	N/A	Please choose the most appropriate course of study.	Undergraduate (Q. 5)	Postgraduate (Q. 6)		Face Validity
5	N/A	What year of undergraduate study are you currently in?	Year			Face Validity
6	N/A	What postgraduate qualification are you undertaking?<br>What year are you currently in?	Masters	PhD	Face Validity	Face Validity
			Year			
7	N/A	Are you a Full-time or Part-time student?	Full-time	Part-time		Face Validity
8	Foubert & Harmon (2013). Higher Education Authority – Eurostudent Survey (adapted)	What is your main field of study?	Humanities and Arts			Face Validity
			Social Sciences, Business and Law			
			Education and teacher training			
			Science, Mathematics and Computing			
			Engineering, Manufacturing and Construction			
			Agriculture and Veterinary			
			Health and Welfare (inc. nursing, health promotion, physiotherapy etc.)			
			Travel, Tourism and Leisure			
			Sports and exercise related			
			Other (please specify)			
9*	N/A	Do you have any long-term illness, health problem or disability that limits your daily activities or work?	Yes (Q. 10)	No (Q. 11)		N/A
10	N/A	Does this prevent you from taking part in sport or exercise?	Yes	No		N/A

**Physical Activity**				
Def: Physical Activity is any movement that results in energy expenditure including job/occupational activities, sports activities (both competitive and recreational), physical conditioning, walking, organised exercise sessions, active transport, or other activities.				
11	Hardie Murphy et al. (2016) CSPPA Plus Young Adult Survey.	Which of these statements best describes your view?	I take enough physical activity to keep healthy.<br>I don't take enough physical activity to keep healthy.	Face Validity
12		How would you rate your level of physical activity compared to other people the same age and sex as yourself?	Much less than others<br>Somewhat less than others<br>About the same<br>Somewhat more than others<br>Much more than others	
Definition: Physical activity can be performed at different intensities:<br>*VIGOROUS intensity physical activity: the effort makes your heart beat much faster and you have to breathe deeper and faster than normal. You will probably sweat.*<br>*MODERATE intensity physical activity: the effort makes you warmer and your heart rate and breathing rate will be faster than normal. You may also sweat a little, but will still be able to carry on a conversation.*				
13	Sport Northern Ireland. (2010). Sport and Physical Activity Survey.	According to current recommendations, what is the minimum amount of moderate to vigorous intensity physical activity that adults should perform to gain health benefits?	Give answer in minutes per day or minutes per week.	Face Validity
Instruction: For the next series of questions, please include ONLY activities of either MODERATE or VIGOROUS intensity.				
14	PACE (Prochaska et al. 2001)	During the LAST 7 DAYS, on how many days were you physically active at a moderate to vigorous intensity for a total of at least 30 minutes per day?	0 – 7 days	Murphy et al. 2017<br>V: r= 0.29<br>R: ICC= 0.70<br>A: 44.5%<br>Compared against accelerometer
15		Over a typical or usual week, on how many days were you physically active at a moderate to vigorous intensity for a total of at least 30 minutes per day?	0 – 7 days	
16*	IPAQ-SF (Craig et al. 2003)	During the last 7 days, on how many days did you do vigorous intensity physical activities for at least 10 minutes at a time?	0 – 7 days<br>'0' response skipped following Q.	Murphy et al. 2017<br>V: r= 0.33<br>R: ICC= 0.52

17		How much time in total did you usually spend on one of those days doing vigorous intensity physical activities?	Hours	Minutes	A: 77.4% Compared against accelerometer
18*		During the last 7 days, on how many days did you do moderate intensity physical activities?	0 – 7 days<br>‘0’ response skipped following Q.		
19		How much time in total did you usually spend on one of those days doing moderate intensity physical activities?	Hours	Minutes	
20*		During the last 7 days, on how many days did you walk for at least 10 minutes at a time? (This includes at university and at home, walking to travel from place to place, and any other walking that you might do solely for recreation, sport, exercise, or leisure.)	0 – 7 days<br>‘0’ response skipped following Q.		
21		How much time in total did you usually spend walking on one of those days?	Hours	Minutes	
22*	Hardie Murphy et al. (2016) CSPPA Plus Young Adult Survey.	During the last 7 days, on how many days did you walk for recreation (i.e. go out for a walk) for at least 10 minutes in your leisure (or free) time?	0 – 7 days<br>‘0’ response skipped following Q.		Face Validity
23		How much time did you usually spend on one of those days walking for recreation?	Hours	Minutes	
24	Hardie Murphy et al. (2016) CSPPA Plus Young Adult Survey.	Which of the following best describes your usual walking pace?	A slow pace<br>A steady pace<br>A fairly brisk pace<br>A fast pace (At least 4 mph)		Face Validity
25*	IPAQ-LF (Craig et al. 2003).<br><br>Sport Northern Ireland (2010). Sport and Physical Activity Survey.	During the last 7 days, on how many days did you cycle for at least 10 minutes at a time? (This includes at college/university and at home, cycling to travel from place to place, and any other cycling that you might do solely for recreation, sport, exercise, or leisure).	0 – 7 days<br>‘0’ response skipped following Q.		Face Validity
26		How much time in total did you usually spend cycling on one of those days?	Hours	Minutes	
27*	Hardie Murphy et al. (2016) CSPPA Plus Young Adult Survey.	How do you usually travel to university? (i.e. what is the longest part of your journey)*	By foot (Q. 29)<br>Bicycle (Q. 29)<br>Car (Q. 28)<br>Bus (Q. 28)<br>Train/ Tram (Q. 28)		Face Validity

			Motorcycle/Scooter (Q. 28)	
28		If you travel by car, bus, train or motorcycle, please give the top 3 reasons why you choose not to walk or cycle.	Too far<br>Too long<br>Too early<br>Too dangerous<br>Not convenient<br>No interest<br>Bad weather<br>No bike available<br>No secure place to lock bike in university<br>Bags are too heavy<br>Need to drop family members elsewhere<br>Other (please specify)	
29		How long does your journey to university usually take?	Hours | Minutes	
30*	Hardie Murphy et al. (2016) CSPPA Plus Young Adult Survey. (adapted)	During the last 7 days, on how many days did you do exercises that may strengthen your muscles, such as push-ups, sit-ups, weight lifting or heavy lifting?	0 – 7 days<br>'0' response skipped following Q.	Face Validity
31		How much time did you usually spend on one of those days doing exercises that may strengthen your muscles?	Hours | Minutes	
32	HE sport participation and satisfaction survey	Do you think that generally you are doing more, less or the same amount of sport and/or recreational physical activity as you did this time last year?	More | Same | Less | Don't Know	Face Validity
33		Now thinking about the future, over THE NEXT 12 MONTHS, would you like to do more sport or recreational physical activity than you do at the moment.	Yes | No | Don't Know	
34		When you selected your university, how important was the university's sporting and physical activity opportunities and facilities in this decision?	1. Not important at all | 10. Extremely Important	
**Determinants of Physical Activity**				
35	DPAQ (Taylor et al. 2013)	For each statement please tick the most appropriate response for you:<br>1. I have NOT previously read information about the current nationally recommended PA guidelines.	1. Strongly disagree<br>2. Disagree a lot<br>3. Disagree a little<br>4. Neither agree or disagree	Taylor et al. 2013 CFA: $X^2(472)$ = 852.3, p<0.01, CFI = 0.933, SRMR =

		2. There is nowhere to do physical activity near me	5. Agree a little<br>6. Agree a lot<br>7. Strongly agree			0.105, RMSEA = 0.042.<br><br>For SASSI, the item with the highest loading in each factor was selected (0.65-0.90)
		3. I feel motivated to do physical activity				
		4. I do not feel confident when doing physical activity				
		5. I don’t seem to have the skills to keep going in physical activity sessions				
		6. I have many negative emotions which prevent me from doing physical activity				
		7. I do not have anyone to do physical activity with				
		8. I think physical activity will change my life for the better				
		9. I tend to plan how my physical activity will happen (e.g. how to get there, kit needed etc)				
		10. I always work around obstacles to physical activity; nothing really stops me				
		11. I would be prepared to give up things I usually do in my leisure time for physical activity				
**Volunteering**						
Definition: The next questions are about sports volunteering (ie. sports voluntary work you have done without receiving any payment except to cover expenses including; organising or helping to run a sports event, campaigning/raising money/providing transport or driving/taking part in a sponsored event/coaching, tuition, mentoring, etc.)						
36*	HE sport participation and satisfaction survey	During the last 4 weeks, have you done any sports voluntary work?	Yes<br>(Q. 37)	No<br>(Q. 38)	Don’t Know<br>(Q. 38)	Face Validity
37		During the last 4 weeks, have you done any of the following activities on a voluntary basis without receiving any payment except to cover expenses? (Please EXCLUDE any time spent solely supporting your own family members.)<br>Indicate number of hours per week for all that apply:	At university<br>Hours per week	Outside university<br>Hours per week		
		1. Coached an individual or team(s) in a sport or recreational physical activity				

		2. Refereed, umpired, or officiated at a sports match or competition				
		3. Performed an administrative or organisational role for a sports club, organisation or event				
		4. Raised funds for a sports club or sport				
		5. Provided transport which helps children or adults take part in a sport (other than family members)				
		6. Provided any other practical help for a sport or recreational physical activity, such as stewarding; helping with sports kit or equipment, or first aid, etc.				
		7. Other (please specify)				
**Coaching**						
38*	HE sport participation and satisfaction survey	Thinking about the last 4 weeks, have you received formal coaching or instruction to improve your performance in any sport or recreational activities? (Do not include informal coaching for example from family members or friends.)*	Yes (Q. 39)	No (Q. 40)	Don't Know (Q. 40)	Face Validity
39		Thinking about the tuition you received from an instructor or coach, did you receive this... Tick all that apply	At a health and fitness facility run by the university			
			In a sports club which is linked to the university			
			At a health and fitness facility not linked to the university			
			Private/freelance instructor not linked to the university			
			Don't know			
			Other (please specify)			
**Sport and Physical Activity Participation**						
40*	HE sport participation and satisfaction survey	Thinking about the last 4 weeks, did you do any sporting or recreational physical activity?	I have not participated in any sport or physical activity either within or outside of my university (Q. 62.)			Face Validity

	– edited to include both in and outside university.		My participation was only through my university or on my university site (Q. 41)	
			My participation was only through organisations and facilities not connected to my university (Q. 51)	
			My participation was both through university and non-university provision (Q. 41)	
41 - 45		Sport/Physical Activity in the past 4 weeks, I have participated in... (max. 5 activities)	**'Inside University' or 'both' responses to Q. 40 are diverted here.**	
		1. Chose Activity	List of 61 sports/activities	
		2. Frequency per week	1 – 3	
			4 – 6	
			7 – 9	
			10 +	
		3. Duration of session	Minutes	
		4. Level of intensity	Light	
			Moderate	
			Vigorous	
		5. Standard	Basic (recreational)	
			Competitive (club level)	
			Elite (regional or international)	
		6. Place of participation	Inside university	
			Outside University	
			Both	
		7. Member of university or external club	University club member	
			External club member	
			Both	
		8. Participate with people	On your own	
			With friends	
			Both	
46	European Commission. (2009). Special	Please rank the top 5 reasons you participate in sport/physical activity (1 = most important, 2 = next most important, etc.)	To make new acquaintances	Face Validity
			To develop skills	
			To have fun	

	Eurobarometer 334 – Sport and Physical Activity.		To improve physical performance		
			Don’t Know		
			To be with friends		
			To improve self-esteem		
			To control weight		
			To improve health		
			For the spirit of competition		
			To counteract the effects of ageing		
			To meet people from other cultures		
			To improve fitness		
			To relax		
			To improve your physical appearance		
			To better integrate into society		
			Other (please specify)		
47	Self-Assessment Review (adapted from the Phase 1 audit tool)	For each of the items below please rate your satisfaction with the provision for sport and physical activity by your university:	1. Extremely dissatisfied	10. Extremely satisfied	Face Validity
		Facilities and playing environment.			
		People and staff organising sport and physical activity.			
		Ease of participating in university sport and physical activity.			
		Opportunities to get sufficient exercise and improve fitness.			
		Opportunities to socialise and feel part of a group or team			
		The coaching and instruction available in my sport and physical activity in university.			
		The value for money I get from the university club membership/fees.			
		The value for money I get from the charges for use of university sport and physical activity facilities and services.			
		Overall satisfaction with sport/exercise provision.			
48*	Hardie Murphy et al. (2016) CSPPA	Since you have begun to study in university, have you taken up any new sports or physical activities?*	Yes (Q. 49)	No (Q. 50)	Face Validity

49	Plus Young Adult Survey.(adapted)	Please indicate the sports/physical activities (max. 5) you have taken up:		
		1. Choose Activity	List of 61 activities/activities	
		2. Place of participation	Inside university	
			Outside university	
			Both	
50*		What is the highest standard that you have achieved in a sport/physical activity in which you are currently participating?	Basic (recreational) (Q. 71)	
			Competitive (club level) (Q. 71)	
			Elite (national, regional, international) (Q. 67)	
			N/A (Q. 71)	
51	HE sport participation and satisfaction survey (adapted)	You participate in sport and/or physical activity but not through your university.<br><br>Please outline your top 3 reasons for not participating through your college/university. (1=most dominant reason; 2=next most dominant reason etc.)	University sport/physical activity does not make me feel welcome by the people who organise of run the activity	Face Validity
			University sport/physical activity does not offer me the right social outlet	
			Don't Know	
			It is not easy to get involved in university sport/physical activity	
			Already involved in a club prior to coming to university	
			University provision is more expensive	
			University facilities are not to my satisfaction in terms of cleanliness	
			It is not easy to make a booking to participate in sport/physical activity at my university	
			It is not as convenient to go to university than to other local facilities in terms of balancing my other commitments such as work, family or study	
			University sport/physical activity does not offer me the appropriate activities or opportunities	
			University sport/physical activity does not offer me the right level of coaching	

			University facilities are not to my satisfaction in terms of quality	
			University sport/physical activity does not offer sport of an informal/non-competitive nature	
			Other (please specify)	
52	HE sport participation and satisfaction survey	What could your university do to encourage you to take up sport or physical activity at your university?	Open Question – Write in option (max. 3)	Face Validity
53 – 57		Sport/Physical Activity in the past 4 weeks, I have participated in outside of university... (max. 5 activities)	**'Outside University' responses to Q.40 are diverted here.**	
		1. Chose Activity	List of 61 sports	
		2. Frequency per week	1 – 3	
			4 – 6	
			7 – 9	
			10 +	
		3. Duration of session	Minutes	
		4. Level of intensity	Light	
			Moderate	
			Vigorous	
		5. Standard	Basic (recreational)	
			Competitive (club level)	
			Elite (regional or international)	
		6. Participate with people	On your own	
			With friends	
			Both	
		7. Member of university or external club	University club member	
			External club member	
			Both	
58	European Commission. (2009). Special Eurobarometer	Please rank the top 5 reasons you participate in sport/physical activity (1 = most important, 2 = next most important, etc.)	To make new acquaintances	Face Validity
			To develop skills	
			To have fun	
			To improve physical performance	
			Don't Know	

	334 – Sport and Physical Activity.		To be with friends		
			To improve self-esteem		
			To control weight		
			To improve health		
			For the spirit of competition		
			To counteract the effects of ageing		
			To meet people from other cultures		
			To improve fitness		
			To relax		
			To improve your physical appearance		
			To better integrate into society		
			Other (please specify)		
59*	Hardie Murphy et al. (2016) CSPPA Plus Young Adult Survey. (adapted)	Since you have begun to study in university, have you taken up any new sports or physical activities?	Yes (Q. 60)	No (Q.61)	Face Validity
60		Please indicate the sports/physical activities (max. 5) you have taken up:			
		1. Choose Activity	List of 61 activities		
61		What is the highest standard that you have achieved in a sport/physical activity in which you are currently participating?*	Basic (recreational) (Q. 71)		
			Competitive (club level) (Q. 71)		
			Elite (national, regional, international) (Q. 67)		
			N/A (Q. 71)		
**Non-participation**<br>'No participation' responses to Q.40 are diverted here.					
62	Sport Northern Ireland (2010). Sport and Physical Activity Survey.<br>HE sport participation and	Please rank the top 3 reasons why you have NOT done any sport or physical activity in the last 4 weeks. (1= most important reason; 2= next most important etc.)	Disability		Face Validity
			I don't feel confident doing sport/physical activity		
			Poor health		
			I'm not good at sport/ physical activity		
			Recent injury, illness, operation or medical reason		

	satisfaction survey (adapted)		Not enough opportunities for me in my local area	
			I am tired during the week	
			No main reason	
			My friends don’t do sport/physical activity	
			Not enough of the right opportunities for me at my university	
			Family commitments	
			I don’t have anyone to do physical activity with	
			It’s too expensive	
			I have no interest	
			Lack of time due to work or study commitments	
			Prefer to spend time doing other activities	
			Other (please specify)	
63	Sport Northern Ireland (2010). Sport and Physical Activity Survey.	Can you tell us how long ago you last participated in sport and / or physical activity or have you never participated?	>4 weeks but < 6 months	Face Validity
			>6 months but < one year	
			>one year but < 5 years	
			5 – 10 years	
			11 – 15 years	
			16 – 20 years	
			20+ years	
			Never participated	
			Don’t Know	
64		Please choose the statement most appropriate to you.	Nothing could encourage me to participate in sports or physical activity.	
			I could be encouraged to participate in sport or physical activity.	
65		Which of the following would encourage you to participate in more sport or physical activity in the future? (Alternatively, which of the following items would help you to be more active?)	Facilities nearer to home/work	
			Better quality facilities	
			Better opening hours	
			Coach/mentor to help me	
			Better information on facilities I could use	

		Please limit your response to 3 per column (i.e. 3 within university and 3 outside of university).	Web or technology support		
			Special programmes		
			Support for my specific needs		
			People to go with		
			Improved transport/ access		
			Help with childcare/crèche facilities		
			Cheaper admission prices		
			Other (please specify)		
66		From your answers above, what is the main item within each category that would encourage you to participate?	Choose one main item per category:<br>1. Within university<br>2. Outside university		
**Elite Athlete Satisfaction**<br>'Elite' responses to Q.50 or Q.61 are diverted here.					
67*	HE sport participation and satisfaction survey	Are you an elite athlete in receipt of a scholarship/bursary from your institution?	Yes (Q. 68)	No (Q. 71)	Face Validity
68		Is this scholarship/bursary sufficient to cover training/competition expenses?	Yes	No	
69		In which sports do you participate as an elite athlete? (max. 3 sports)	List of 57 activities		
70		Please rate your satisfaction with the provision for elite athletes in your university.	1. Extremely dissatisfied	10. Extremely satisfied	
**Related Health Behaviours**					
71	Morgan et al. (2008). Survey of Lifestyle, Attitudes & Nutrition in Ireland.<br><br>Foubert & Harmon (2013). Higher Education	How often do you have a drink containing alcohol?	Never		Face Validity
			Monthly or less		
			2 – 4 times a month		
			2 – 3 times a week		
			4 or more times a week		
72		Do you now smoke every day, some days, or not at all?	Every day		
			Some days		
			Not at all		
73		Have you ever taken non-prescribed/ recreational drugs?	No		
			Yes but stopped		

	Authority – Eurostudent Survey (adapted)		Yes and still do	
74	Milton et al. 2011	In the PAST 7 DAYS, on how many days have you done a total of 30 minutes or more of physical activity, which was enough to raise your breathing rate?<br>(This may include sport, exercise, and brisk walking or cycling for recreation, or to get to and from places, but should not include housework or physical activity that may be part of your job.)	0 – 7 days	Murphy et al. 2017<br>V: r= 0.30<br>R: ICC= 0.69<br>A: 45.2%<br>Compared against accelerometer
75	Marshall et al. 2010	Please estimate how many minutes you spend sitting each day in the following situations:	Weekday (minutes) / Weekend (minutes)	Marshall et al. 2010<br>Weekday<br>V: r= 0.21 - 0.74<br>R: ICC= 0.62 - 0.86<br><br>Weekend<br>V: r= 0.13 - 0.64<br>R: ICC= 0.59 - 0.72
		1. While attending lectures and classes		
		2. While studying		
		3. While travelling to and from places		
		4. While at work		
		5. While watching television		
		6. While using a computer at home		
		7. While using a smart phone/ tablet		
		8. In your leisure time, not including television		
76	Morgan et al. (2008). Survey of Lifestyle, Attitudes & Nutrition in Ireland.	How often do you eat convenience food (i.e. fast food or 'takeaways': e.g. Chinese, Indian, pizza, burgers, chips)	Daily<br>4 – 6 times a week<br>1 – 3 times a week<br>Less than once a week<br>Never	Face Validity
77		How often do you prepare food from fresh ingredients rather than pre-prepared food?	Daily<br>4 – 6 times a week<br>1 – 3 times a week<br>Less than once a week<br>Never	
78	Hardie Murphy et al. (2016) CSPPA Plus Young Adult Survey.	Do you think your body is…?	Much too thin<br>A bit too thin<br>About the right size<br>A bit too fat	Face Validity

			Much too fat	
79	Sport Northern Ireland (2010). Sport and Physical Activity Survey.	Over the past 12 months, would you say your health has been?	Very good<br>Good<br>Average<br>Poor<br>Very poor<br>Don't Know	Face Validity
80		In general, how happy would you say you are?<br>Please rate on a scale of 1 – 10, where 1 is "Extremely unhappy" and 10 is "Extremely happy".	1 – 10	
81	Mental Health Index (MHI-5) Ware et al. 1992	How much of the time during the past 4 weeks have you…<br>1. Been a nervous person<br>2. Felt so down in the dumps that nothing could cheer you up<br>3. Felt calm and peaceful<br>4. Felt downhearted and blue<br>5. Been a happy person	Options include:<br>- All of the time<br>- Most of the time<br>- A good bit of the time<br>- A little of the time<br>- None of the time	Houghton et al. 2010<br>V: r = -0.65 - -0.71<br>IC: α = 0.783<br>R: r = 0.403<br>Compared against other self-report instruments.
**Demographics**				
82	Foubert & Harmon (2013). Higher Education Authority – Eurostudent Survey	Please estimate the gross (before tax) annual income of your family household.<br>Indicate in € or £.	Less than 20,000<br>20,001 – 35,000<br>35,001 – 50,000<br>50,001 – 70,000<br>70,001 – 90,000<br>>90,000<br>Don't Know	Face Validity
83	Higher Education Sports Participation and Satisfaction	What is the highest level of education your parent(s)/ guardian(s) have attained to date?	Mother / Father<br>Some primary (not complete)<br>Complete primary or equivalent<br>GCE O level<br>GCE A level	Face Validity

	Survey-edited from UK version		Intermediate/Junior/ Group certificate<br>Leaving certificate or equivalent<br>Diploma/Certificate<br>Primary degree<br>Postgraduate/ Higher Degree<br>Don't Know<br>Other (please specify)	
84		Are your parent(s) / guardian(s) currently employed?<br>If one or both of your parents are deceased, please tick Not Applicable (NA)	Yes<br>No<br>Don't Know<br>N/A	
85*	Foubert & Harmon (2013). Higher Education Authority – Eurostudent Survey (adapted)	Do you work as well as study at university?	Yes – Working full-time<br>Yes – Working part-time<br>No (Skip next Q.)	Face Validity
86		Please indicate the numbers of hours you work per week.	Hours per week	
87	Higher Education Sports Participation and Satisfaction Survey-edited from UK version	How tall are you?<br>Use either centimetres or feet/inches.	Height in Feet/Inches<br>Height in Centimetres	Face Validity
88		What weight are you?<br>Use either kilograms or stone/pounds	Weight in stone/pounds<br>Weights in kilograms	
89		Is the accommodation you live in during term time...?	University halls of residence (on campus)<br>University halls of residence (off campus)<br>Rented privately<br>Your family home<br>Other (please specify)	
90		Please tick as appropriate…	I spend most weekends (i.e. 3 per month) at my university accommodation during term time.<br>I spend about half my weekend at my university accommodation during term time.<br>I usually go home at the weekends.	
91	N/A	Are you married?	Yes | No	Face Validity

92	N/A	Do you have children?	Yes	No	Face Validity
93	N/A	In which country were you born?	Ireland – Republic<br>Ireland – Northern Ireland<br>Other (please specify)		Face Validity
94	N/A	Which of these ethnic groups do you consider you belong to?	White<br>Black<br>Asian<br>Mixed or multiple ethnic groups<br>Other (please specify)		Face Validity
**End/Re-contact**					
95	N/A	Would you like to be contacted to possibly take part future physical activity and health measurements?	Yes	No	N/A
96	N/A	Would you be interested in taking part in interviews/focus groups as part of this study?	Yes	No	N/A
97	N/A	To thank you for participating in this survey, we would like to offer you the chance to be included in a prize draw. Would you like to be included in the prize draw?	Yes	No	N/A
98	N/A	If you answered yes to any of the questions above please enter your email and / or mobile number in the boxes provided.<br>These details will only be used to contact you if you are selected as a potential participant for the physical activity and health measurements, the interview process or if you are a prize draw winner.<br>Your details will not be used for any other purpose and will remain strictly confidential.	Open Question		N/A
**Thank You**					
Thank you for your participation in this survey.<br>*To submit your data please click the 'SUBMIT' button below.*					

*= denotes a filter question, where responses determine the path to the following question. For these the following question can be seen in brackets beside the responses; R= reliability; V= validity; CFI= confirmatory factor analysis; A= level of agreement.
